# An environmental type-recessiveness framework for the spatial coupling of environmental heterogeneity and ICH recessiveness in Guizhou’s ethnic minority villages, China

**DOI:** 10.1371/journal.pone.0360100

**Published:** 2026-10-22

**Authors:** Rui Guo, Ancan Yao

**Affiliations:** School of Architecture and Urban Planning, Guizhou University, Guiyang, Guizhou, China; International Islamic University Malaysia, MALAYSIA

## Abstract

The relationship between environmental heterogeneity and intangible cultural heritage (ICH) remains insufficiently explored at the regional scale. Using data from 312 ethnic minority villages and 305 ICH items in Guizhou, China, this study develops an Environmental Type–Recessiveness Cultural Adaptation (ERC) framework to examine associations between environmental conditions and ICH distribution patterns. Four environmental variables were integrated into five environmental types using K-means clustering. ICH recessiveness was operationalized as a three-dimensional composite index comprising observability, carrier explicitness, and environmental association. Expert scoring exhibited strong reliability. Results reveal clear associations between environmental settings and ICH recessiveness: moderate recessiveness predominates across the province; high-elevation, high-elevation environments with large distance to water systems tend to host more recessive forms of heritage, whereas low-relief and highly accessible landscapes are associated with lower recessiveness. These findings indicate that environmental differentiation is spatially coupled with systematic variation in ICH recessiveness. Such spatial correspondence is consistent with processes of socio-ecological adaptation rather than environmental determinism. The ERC framework provides a standardized and reproducible quantitative approach for comparative analysis of ICH across heterogeneous landscapes, offering a transferable tool for heritage research in mountainous and ethnically diverse regions. Overall, the observed west-to-east gradient in ICH recessiveness further supports the role of socio-ecological adaptation in shaping ethnic heritage patterns in mountainous environments.

## Introduction

The concept of cultural landscapes, formally recognized by UNESCO in 1992 [1], frames the dynamic socio-ecological interplay between humans and nature. Extensive research has demonstrated that environmental differentiation plays an important role in cultural formation and adaptation [2,3], shifting landscape studies from abstract symbolism to tangible socio-ecological systems. Globally, environmental heterogeneity shapes distinct cultural landscapes, such as Andean terraces and Southeast Asian agroforestry [4–6]. These examples illustrate that landscapes are co-produced through long-term human-environment adaptation, rather than static backdrops or isolated artifacts.

The 2003 UNESCO Convention emphasizes that intangible cultural heritage (ICH)—including oral traditions, rituals, and ecological knowledge—is deeply embedded in specific environmental and cultural contexts [7]. Recent scholarship has advanced the concept of recessive cultural landscapes, defined as less visible and environmentally contingent cultural practices that differ from tangible monumental forms of heritage [8]. Recessive ICH, such as Japanese satoyama ceremonies and Andean agricultural knowledge systems, is characterized by strong environmental dependence and limited material expression [9,10]. Although case studies have linked ICH recessiveness to environmental embeddedness, systematic spatial analyses of environment–ICH relationships remain limited at broader regional scales [11–14].

GIS and spatial analytical techniques have substantially advanced quantitative investigations of culture–environment relationships, moving beyond descriptive studies. Recent studies have increasingly employed spatial statistics, geospatial modeling, and environmental analytical frameworks to explore cultural heritage across multiple spatial scales. Nationwide comparative analyses of tangible and intangible cultural heritage have adopted geodetector tools paired with a suite of geospatial statistics to unpack divergent natural and socioeconomic driving forces, with spatial heterogeneity identified as a core feature shaping national heritage distribution patterns [15]. Further methodological progress in multi-scale spatial modeling has made it possible to disentangle scale-varying impacts of environmental and human factors on intangible cultural heritage, confirming that distinct predictors exert influence over different geographic extents [16]. Outside heritage-focused research, integrated landscape ecology workflows have been constructed to quantify coupled human–ecosystem interactions, offering actionable spatial frameworks for regional sustainable land management [17]. Taken together, these methodological innovations have elevated terrain, distance to water systems and topographic metrics to core variables within quantitative cultural landscape research. Meanwhile, parallel theoretical progress in intangible heritage studies has established a tripartite conceptual system to separate recessive from overt cultural practices, built around the dimensions of public observability, material carrier clarity, and environmental dependency, laying preliminary theoretical groundwork for numerical evaluation of how deeply heritage is embedded within local ecological contexts.

In China, research on cultural landscapes has primarily focused on tangible expressions such as architecture, settlement morphology, and agricultural systems [18–22], while studies of ICH have remained largely within ethnographic or folkloric traditions [23,24]. This disciplinary separation has limited systematic understanding of how ecological gradients correspond to variation in intangible heritage. Guizhou Province, located on the Yunnan–Guizhou Plateau, hosts the largest number of ethnic minority villages in China and sustains remarkable cultural diversity among groups such as the Miao, Dong, Buyi, and Shui. Yet most research on Guizhou’s minority landscapes remains limited to descriptive analyses or case-based ethnographies, lacking spatially explicit and quantitative perspectives. This gap has become increasingly critical amid rapid urbanization and tourism development, which are transforming minority cultural landscapes and threatening recessive traditions [25]. In response, the Guizhou provincial government issued *The Implementation Plan for Supporting the High-Quality Development of Culture and Tourism in Guizhou (2022)* [26], advocating a categorized approach to safeguarding ICH and the villages that sustain it within an integrated culture–tourism framework. These initiatives highlight the urgent need for spatially grounded research examining how ecological differentiation corresponds to variations in ethnic minority cultural landscapes in Guizhou as reflected in patterns of cultural recessiveness.

Despite these methodological advances, several important analytical challenges remain unresolved. First, although recent spatial studies have substantially improved our understanding of the distribution patterns and influencing factors of cultural heritage, they pay limited attention to how differentiated environmental settings correspond to variation in ICH recessiveness. Second, environmental influences are typically examined using individual explanatory variables rather than integrated environmental regimes that capture the combined effects of multiple ecological characteristics. Third, although the concept of cultural landscape recessiveness has received increasing attention, a standardized analytical framework integrating environmental typology with multidimensional recessiveness assessment has not yet been established. Consequently, the spatial coupling between environmental heterogeneity and ICH recessiveness remains insufficiently understood. Building on these unresolved limitations, this study hypothesizes that environmental regimes align with recurrent spatial differences in ICH recessiveness. Specifically, high-elevation settings with large distance to water systems are associated with greater recessiveness; accessible, low-relief landscapes exhibit lower recessiveness; and intermediate environments display moderate recessiveness.

In response to these limitations and the practical need for spatially grounded heritage assessment, this study examines how environmental differentiation is spatially coupled with differentiated variation of ICH among ethnic minority villages in Guizhou. Specifically, the study aims to (1) analyze the spatial distribution of 312 ethnic minority villages; (2) classify village environments into distinct types using elevation, topographic relief, slope aspect, and distance to water systems; and (3) construct a composite recessiveness indicator to assess 305 ICH items and explore their distribution across environmental types. By integrating environmental classification with measurable recessiveness differentials, the ERC framework is established as a standardized analytical tool for interpreting spatial variability in ICH expression.

In this study, ICH recessiveness is conceptualized as a multidimensional socio‑ecological construct referring to the extent to which intangible cultural practices are environmentally embedded, tacitly transmitted, and materially unobtrusive. It reflects a continuum from highly visible, publicly accessible heritage to hidden, context‑dependent cultural forms. Importantly, recessiveness is employed here as a relational, descriptive metric of visibility and environmental embeddedness, not an assessment of inherent heritage value. This conceptualization builds on recent heritage‑science research on hidden cultural landscapes [27,28]. Unlike conventional frameworks that prioritize tangible value assessments and view the environment as a passive backdrop, the ERC framework provides an analytical workflow that integrates quantified environmental types with a neutral three‑dimensional recessiveness metric, shifting the focus from normative heritage evaluation to socio‑ecological pattern analysis.

## Objects and methods

### Research objects

Guizhou Province, located on the Yunnan-Guizhou Plateau in southwestern China, is characterized by highly complex topography, with more than 100 peaks exceeding 2,000 m in elevation. The study area encompasses 312 ethnic minority villages selected from the *List of Chinese Ethnic Minority Villages with Distinctive Features* (covering three batches in total). To characterize the environmental context of these villages and support the analysis of environmental differentiation, key variables-including elevation, topographic relief, slope aspect, and distance to major water systems—were compiled from multiple geospatial datasets, including the *National Geospatial Information Public Service Platform* [29], the *ALOS Research and Application Project* [30], and the Global Surface Water Dataset. The basic environmental analysis data for all 312 villages are provided in S1 Table. In terms of spatial resolution, the elevation, topographic relief, and slope aspect were derived at 12.5 m resolution, while the original water system data have a resolution of 30 m. All datasets were processed and vectorized to construct a quantitative spatial database using ArcGIS 10.2.

Within these 312 villages, 305 ICH items were identified from the *Guizhou Provincial* ICH *List*. Following the conceptual framework established in previous scholarship, this study conceptualizes the cultural landscape as an ecological–cultural system in which ICH practices are embedded and expressed. Accordingly, ICH practices nurtured within ethnic minority villages are not merely viewed as components of the landscape but as key indicators through which adaptive relationships between culture and environment can be interpreted, with the villages themselves serving as the primary carriers of these landscape expressions. The integration of spatial and cultural datasets provides an empirical foundation for examining the spatial differentiation of cultural landscape carriers, identifying environmental types, and elucidating the mechanisms linking environmental context to cultural landscape characteristics in ethnic minority villages in Guizhou.

## Methods

### ERC framework

To explore spatial linkages between ecological variation and ICH expression, the ERC approach is constructed via a standardized indicator-based workflow. The framework comprises three analytical stages (Fig 1). First, environmental typology integrates settlement spatial distribution analysis with multivariate environmental clustering to characterize ecological differentiation among ethnic minority villages. Second, ICH recessiveness is operationalized as a composite analytical construct reflecting the degrees of cultural visibility, material expression, and environmental embeddedness. Third, environmental typology and recessiveness patterns are jointly analyzed to interpret how environmental gradients relate to the spatial characteristics of cultural landscapes. Together, these stages enable the systematic assessment of environment-heritage coupling.

**Fig 1:**
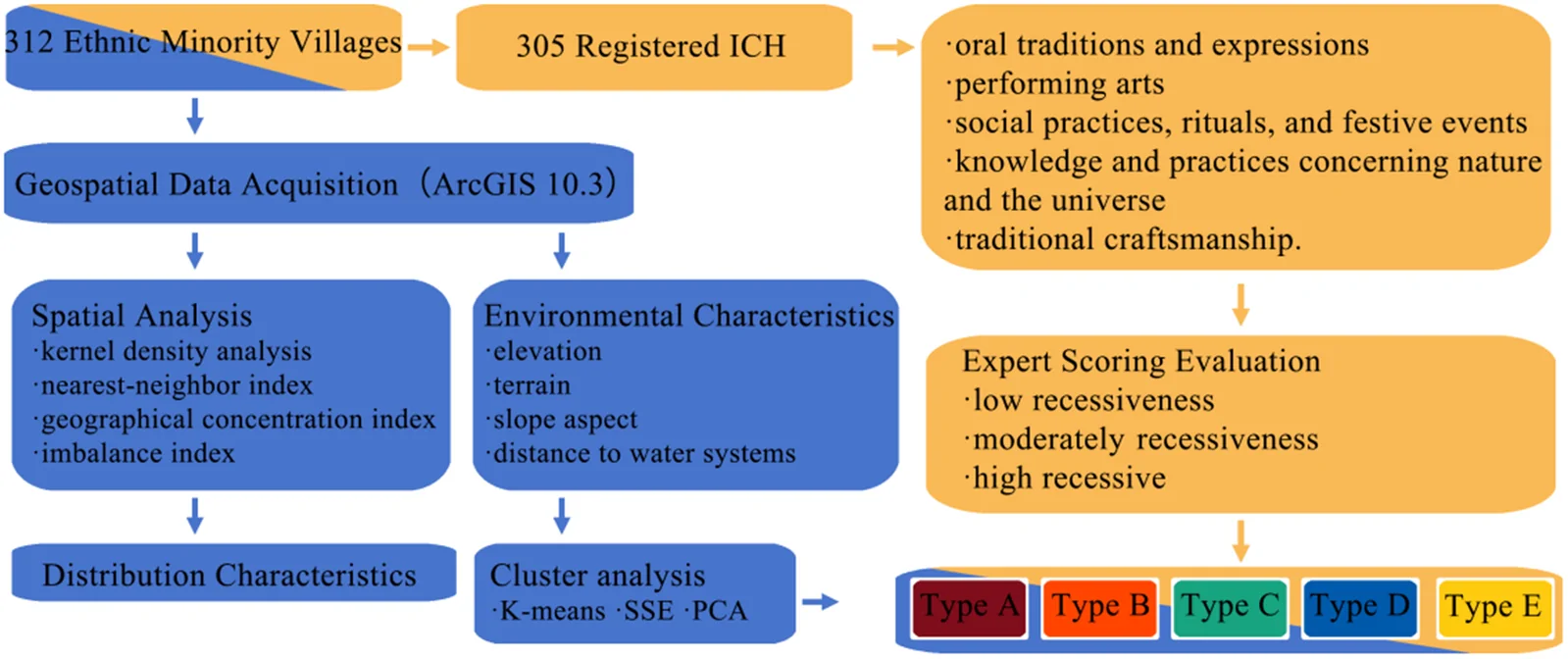
ERC framework linking environmental differentiation and cultural landscape recessiveness. The framework integrates standardized environmental classification and a composite recessiveness index to enable spatial comparison of environment–heritage relationships.

### Spatial distribution analysis

At the provincial scale, the spatial distribution of ethnic minority villages was examined using four complementary spatial indices (Fig 1). Kernel density analysis (KDA; Eq. 1) was applied to generate a continuous density surface through weighted smoothing, allowing visualization of agglomeration intensity and regional spatial trends [31]. The nearest-neighbor index (Eq. 2) assessed whether village distribution deviated from randomness by comparing observed and expected mean inter-settlement distances [32]. The geographic concentration index (Eq. 3) quantified the degree of spatial concentration, with values ranging from 0 (uniform distribution) to 100 (complete centralization). In addition, the imbalance index (Eq. 4) evaluated distributional equilibrium across administrative units, where values approaching 1 indicate increasing spatial imbalance.

These indices collectively characterize the macro-scale spatial organization of ethnic minority settlements, providing a spatial context for subsequent environmental classification.

### Environmental clustering

To identify regionally differentiated environmental contexts of ethnic minority villages, four ecological indicators were selected: elevation, representing climatic conditions and vertical resource stratification; topographic relief, indicating terrain ruggedness and settlement constraints; slope aspect, influencing solar radiation, microclimate, and agricultural suitability; and distance to water systems, reflecting access to subsistence resources and water-access constraints. These indicators capture the fundamental environmental conditions governing long-term settlement persistence. Environmental typologies were delineated using K-means clustering applied to the 312 villages [33]. The optimal number of clusters was determined using the elbow criterion, silhouette scores, explained variance, and principal component analysis (PCA)-based visual inspection. The clustering structures and PCA-based comparisons among alternative cluster solutions are illustrated in Fig 2. As shown in Table 1, the five-cluster solution achieved the highest silhouette score (0.56) among the tested solutions while explaining 83.2% of the total variance. Together with PCA-based visual inspection and cluster interpretability, these results supported the selection of five clusters for subsequent analysis.

**Table 1:** Validation metrics for determining the optimal number of environmental clusters.

Number of clusters (k)	Sum of Squared Errors	Silhouette score	Variance explained (%)
2	892.6	—	—
3	754.3	0.42	62.3
4	618.9	0.49	74.5
5	523.7	0.56	83.2
6	512.2	0.53	85.1

Note: k = the preset number of K-means clusters; Sum of Squared Errors (SSE) is a dimensionless metric measuring intra-cluster deviation; Silhouette score ranges from −1–1, where higher values indicate better clustering separation; Variance explained (%) represents the proportion of total environmental variable variation captured by clusters. All values in this table are statistical calculation results, not counts of villages or ICH items, and no cell values correspond to percentage proportions of samples.

**Fig 2:**
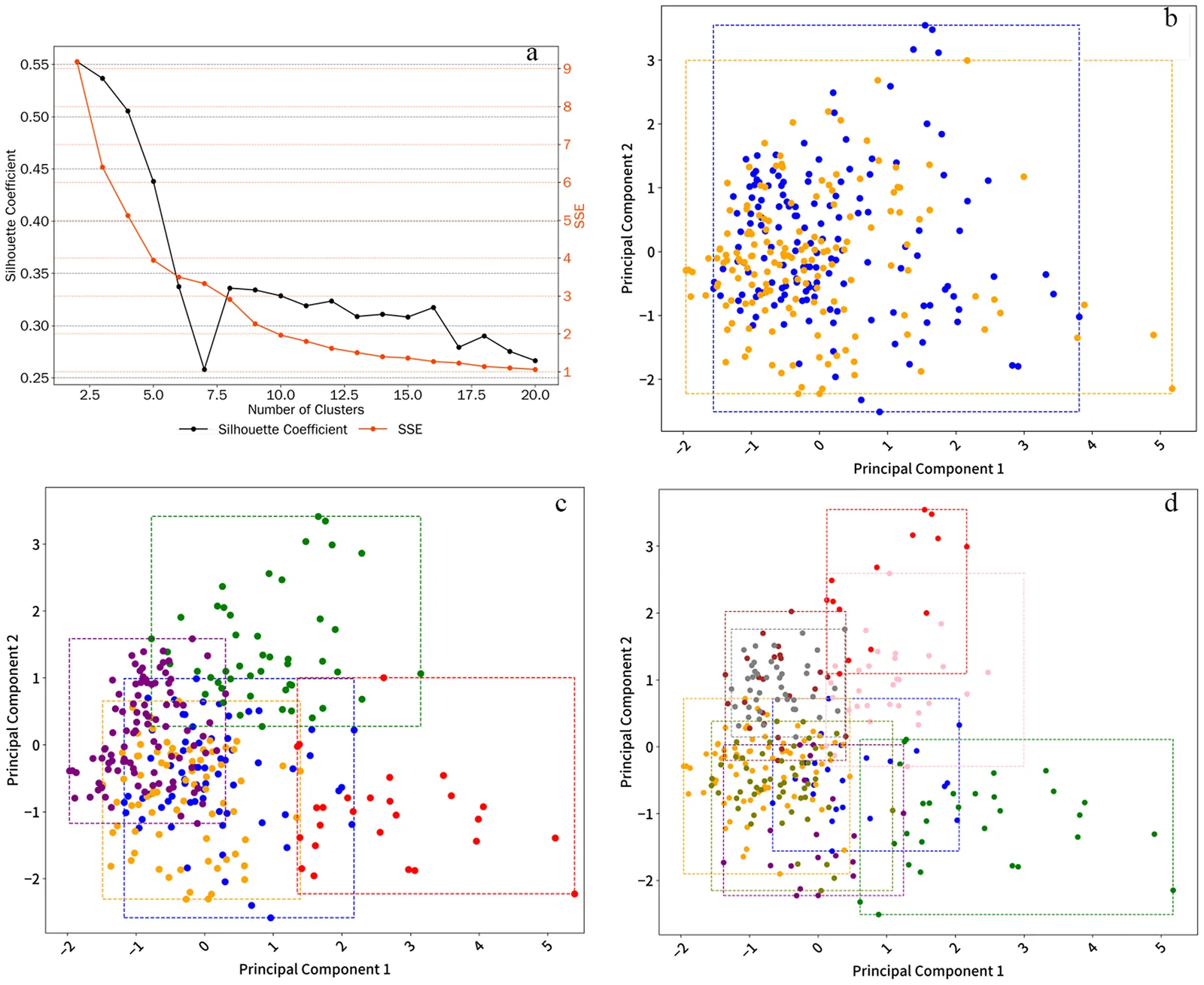
Environmental clustering structures and PCA-based visualization of alternative cluster solutions. Environmental variables were standardized using min–max normalization prior to K-means clustering. (a) Results of the selected five-cluster solution (k = 5); (b–d) PCA projections illustrating clustering stability under different cluster numbers (k = 2, 5, and 9).

### ICH data integration

To examine interactions between environmental differentiation and ICH expression, 305 ICH items associated with the 312 ethnic minority villages were compiled from the *Guizhou Provincial ICH List*. In accordance with *The Convention for the Safeguarding of the ICH*, all items were classified into five UNESCO-defined domains: oral traditions and expressions; performing arts; social practices, rituals, and festive events; knowledge and practices concerning nature and the universe; and traditional craftsmanship.

Because the number of villages and registered ICH items did not correspond on a one-to-one basis, all 312 villages were retained as environmental observation units. ICH items were spatially assigned to villages based on official documentation. Villages without registered ICH entries were retained as null environmental cases for spatial distribution and environmental clustering analyses, while being excluded from recessiveness scoring and gradient-based comparisons. This approach allows the ERC framework to represent the broader environmental distribution of both heritage-associated and non-registered villages, rather than limiting the analysis exclusively to heritage-positive cases.

### Recessiveness operationalization and contextual expert input

ICH recessiveness is measured as a multidimensional index capturing cultural visibility, material expression, and environmental embeddedness. Three measurable dimensions are defined: observability (Obs), carrier explicitness (Car), and environmental association (Env). These dimensions are theoretically grounded in heritage science and cultural landscape research on visibility, material codification, and ecological embeddedness of ICH practices. Observability reflects the degree of public visibility and perceptual accessibility, distinguishing highly exposed heritage forms from context-dependent ones. Carrier explicitness captures the extent of material codification and institutional anchoring, indicating whether practices are tacitly transmitted or formally represented. Environmental association operationalizes the degree to which ICH practices depend on specific ecological settings and localized environmental conditions. Together, these three dimensions capture the extent to which ICH practices remain tacit, weakly codified and materialized, and environmentally embedded, constituting the core analytical meaning of recessiveness within the ERC framework. In this study, recessiveness does not imply cultural inferiority or concealment but refers to the relative degree to which ICH practices remain environmentally embedded, weakly codified and materialized, and less publicly observable within standardized comparative analysis. This framework provides a standardized basis for measuring variation in ICH embeddedness across environmental settings.

A structured expert evaluation was conducted using a multidisciplinary panel of 10 specialists in ICH studies, cultural geography, ethnology, landscape conservation, quantitative spatial analysis, and local heritage administration. Panel members were recruited based on at least five years of relevant professional experience, direct familiarity with ethnic minority heritage contexts in Guizhou, and absence of conflicts of interest. To protect expert confidentiality, personal demographic information was anonymized; therefore, detailed characteristics such as gender, regional representation, and institutional affiliation are not reported. Before formal evaluation, a detailed scoring manual was developed specifying conceptual definitions, five‑point scale anchors (1 = lowest recessiveness to 5 = highest recessiveness), boundary conditions, and reference cases (Fig 3). A pilot calibration exercise was conducted to align interpretation criteria across panel members.

**Fig 3:**
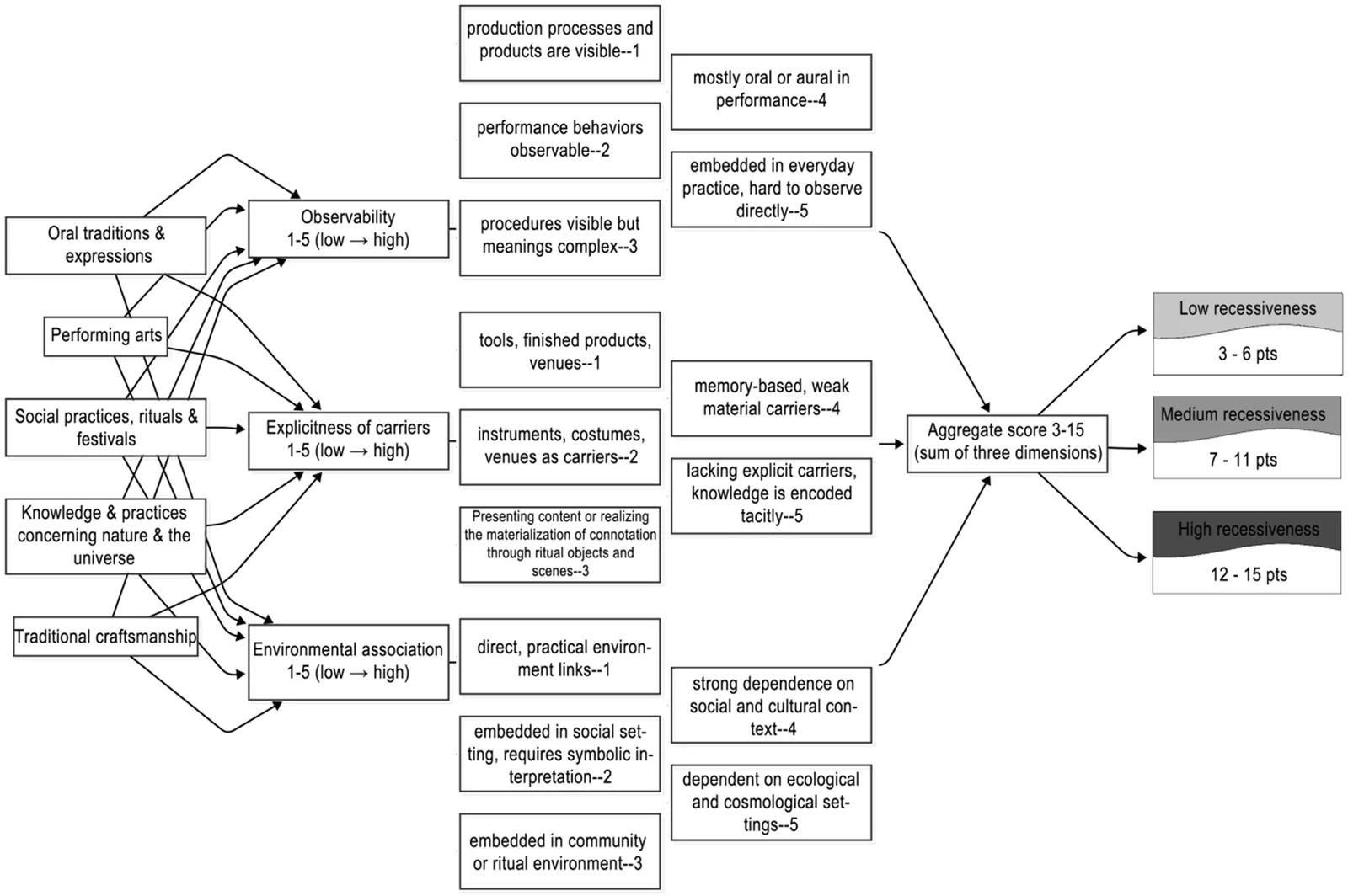
Evaluation of ICH recessiveness levels based on a composite index integrating observability, carrier explicitness, and environmental association.

Each of the 305 ICH items was scored independently by the ten experts across the three recessiveness dimensions. The raw expert scoring matrix is provided in S2 Table. To mitigate subjective bias and improve scoring consistency, items exhibiting inter-rater score ranges of ≥2 points were reviewed through collective discussion to clarify interpretive boundaries and ensure consistent interpretation of the evaluation criteria. Such cases accounted for 7.2% of the dataset. Final dimensional scores were calculated as the arithmetic mean across raters, thereby reducing individual variation and enhancing measurement stability. The reliability and consistency of the scoring system were assessed using Cronbach’s α [34], and the intraclass correlation coefficient (ICC) [35]. The resulting values indicated strong internal consistency and substantial inter-rater agreement, supporting the reliability of the expert scoring framework.

The summed score of the three dimensions represents overall recessiveness. Equal weighting was adopted because observability, carrier explicitness, and Environmental association were conceptualized as complementary and equally important dimensions of ICH recessiveness, and no theoretical or empirical basis existed for assigning differential weights. For cross-environmental comparison, composite scores were classified into three categories: low recessiveness (≤4.5), medium recessiveness (4.5–8.0), and high recessiveness (>8.0). These thresholds were determined using Jenks natural breaks optimization, a data‑driven method that minimizes within‑group variance and maximizes between‑group separation.

### Reliability assessment

Key reliability metrics were calculated to validate the recessiveness measurement. Cronbach’s α coefficients for the three dimensions ranged from 0.832 to 0.859, indicating strong internal consistency. Inter-rater agreement (ICC(2,k)) ranged from 0.832 to 0.858, reflecting substantial expert consensus. Inter-expert correlations are visualized in Fig 4. Overall, the framework achieved satisfactory reliability for spatial analysis.

**Fig 4:**
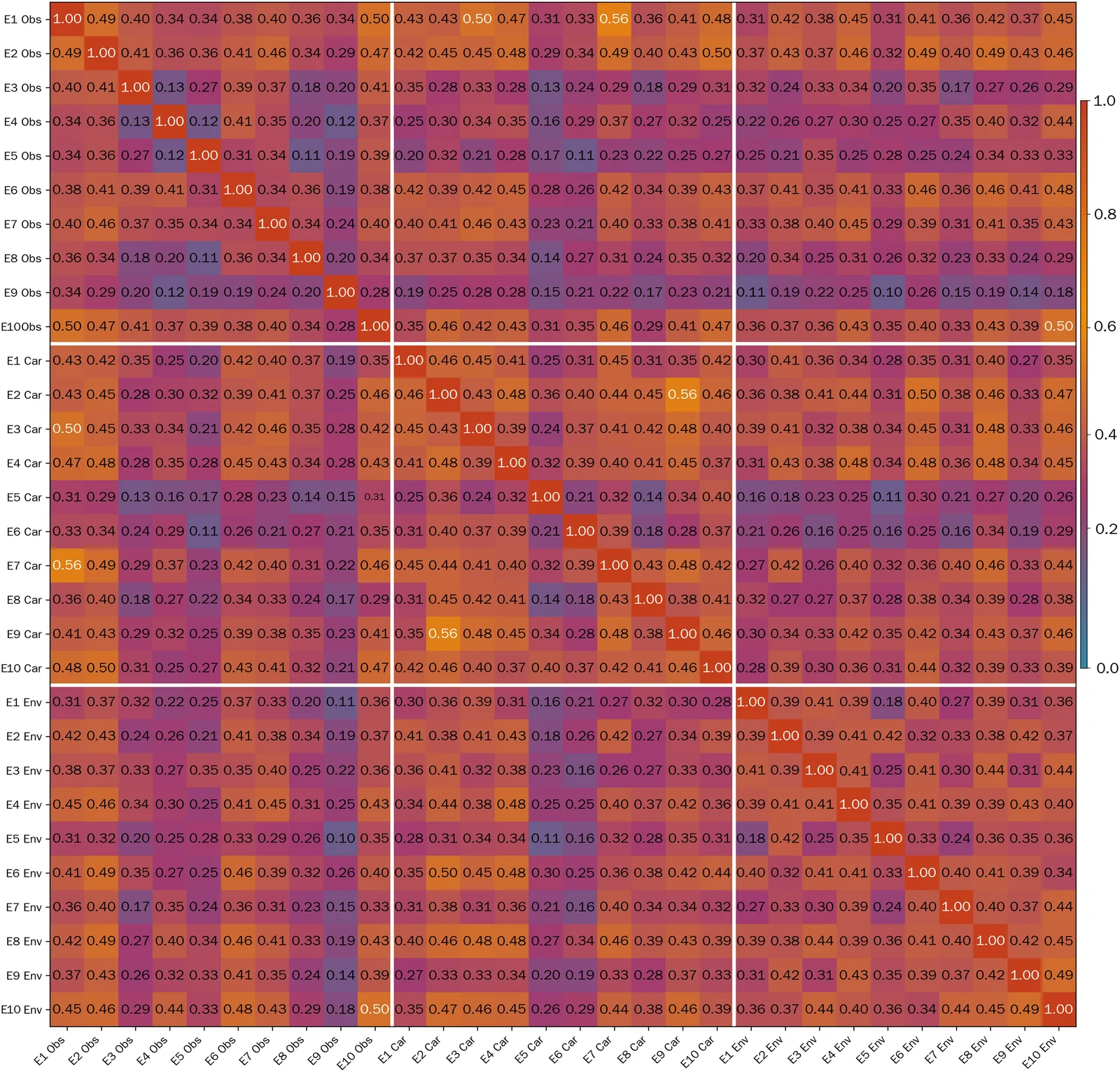
Correlation heat map of the three ICH recessiveness dimensions. Scores were standardized prior to analysis. Colour gradients represent correlation strength, ranging from weak (light colours) to strong (dark colours).

### Dimensional patterns and gradient analysis

Gradient analysis was performed using villages as analytical units, examining recessiveness scores across four environmental gradients: elevation, topographic relief, slope aspect, and distance to water systems. This analysis quantifies patterned environment–ICH associations and identifies consistent spatial correspondences without implying causal inference.

All analyses were conducted using standardized GIS workflows in ArcGIS 10.2. Environmental and recessiveness datasets were processed consistently across all villages, and the robustness of K-means clustering was evaluated via multiple initializations and supplementary validation metrics.


$$\begin{array}{c} f\left(x\right)=\frac{\text{1}}{nh^{\text{2}}}\sum_{i=1}^{n}K\left(\frac{d_{i}}{h}\right) \end{array}$$ (1)

where *n* is the total number of villages, *h* denotes the bandwidth, *d*_*i*_ represents the distance between location *x* and village *i*, and *K* (·) is the kernel function.


$$\begin{array}{c} \text{R}=\frac{{\overset{―}{d}}_{obs}}{{\overset{―}{d}}_{exp}}\text{\textasciitilde{}}\;\text{\textasciitilde{}}{\overset{―}{\text{d}}}_{\text{exp}}=\frac{\text{1}}{2\sqrt{n/A}} \end{array}$$ (2)

where ${\overset{―}{d}}_{obs}$ is the observed mean nearest-neighbor distance and ${\overset{―}{d}}_{\exp}$ is the expected mean distance under spatial randomness, *n* represents the number of villages *A* and denotes the total study area (km²).


$$\begin{array}{c} G=100\times \sqrt{\sum_{i=1}^{m}{\left(\frac{x_{i}}{T}\right)}^{\text{2}}} \end{array}$$ (3)

where *x*_*i*_ denotes the number of villages in administrative unit *i*, *T* is the total number of villages, and *m* is the total number of administrative units. The multiplication by 100 standardizes the index to a 0–100 scale, where higher values indicate stronger spatial concentration.


$$\begin{array}{c} S=\frac{\text{1}}{\text{2}}\sum_{i=1}^{m}\left|\frac{x_{i}}{T}-\frac{\text{1}}{m}\right| \end{array}$$ (4)

where *x*_*i*_, T, *m* are defined as above. The index ranges from 0 (perfectly even distribution) to 1 (maximum imbalance), reflecting the deviation from uniform spatial allocation.

### Ethics statement

Expert participants involved in the scoring process provided verbal informed consent prior to participation. Participation was voluntary and anonymous, and no sensitive personal data were collected. Verbal consent was documented by the research team during the evaluation process. The study involved only non-interventional expert-based assessment and did not involve vulnerable human subjects; therefore, formal ethical approval was not required under institutional research guidelines.

## Results

### Spatial distribution characteristics

Guizhou Province contains 17,935 administrative villages, of which 312 are classified as ethnic minority villages. Based on the spatial indices described in *Spatial distribution analysis* subsection within the Objects and methods section, the nearest-neighbor index indicates a clustered distribution of ethnic minority villages. The observed mean nearest-neighbor distance is 0.096, compared with a theoretical expected distance of approximately 0.14, yielding a nearest-neighbor ratio of 0.69, which indicates a clustered spatial pattern at the provincial scale.

Using Equation (3), the geographic concentration index (G) for ethnic minority villages is calculated as 5.79, which is relatively low within the 0–100 range, suggesting limited large-scale centralization despite local clustering. The imbalance index (S), calculated using Equation (4), is 0.014, indicating a relatively even distribution across administrative regions. In contrast, for all administrative villages in Guizhou, the nearest-neighbor ratio is 0.74, the geographic concentration index is 12.70, and the imbalance index is 0.24, reflecting a more centralized and uneven spatial structure.

Kernel density analysis further illustrates these differences. Non-ethnic minority villages display multiple high-density agglomerations, particularly in the northeastern and northwestern regions of the province. Ethnic minority villages exhibit a contrasting pattern, with primary concentrations in the southwestern part of Qiandongnan Miao and Dong Autonomous Prefecture and the northeastern portion of Qiannan Buyi and Miao Autonomous Prefecture. Overall, ethnic minority villages exhibit pronounced spatial heterogeneity, characterized by small-scale clustering embedded within a relatively dispersed provincial framework.

### Environmental characteristics and typological structure of ethnic minority villages

The environmental settings of the 312 ethnic minority villages were characterized using four indicators: elevation, topographic relief, slope aspect, and distance to water systems.

Elevation spans from 252 m to 2,678 m, with a total range of 2,426 m. The mean elevation is 936.26 m (SD = 403.66 m), indicating substantial vertical heterogeneity. Approximately 68% of villages are located between 500 and 1,000 m, while the upper 10% exceed 1,200 m and are concentrated in mountainous southwestern regions. Lower-elevation villages (<400 m) are primarily distributed in northeastern river valleys.

Slope aspect spans nearly the full directional spectrum (0°–357.51°), with a mean of 192.23° and a median of 198.36°. Northwest-facing slopes are most frequent (n = 54), whereas northeast-facing slopes are least common (n = 20). Variability differs across aspects, with high dispersion for northeast-facing slopes (CV = 0.31) and strong clustering for northwest-facing slopes (CV = 0.04), indicating uneven orientation preferences across the landscape.

Topographic relief ranges from 2.0 to 157.0, with a mean of 98.3 and a median of 94.0. The proximity of mean, median, and mode values indicates a relatively balanced distribution centered on moderate relief. Only two extreme values (0.64%) fall outside the ± 3σ range, suggesting that highly rugged terrain is uncommon among the sampled villages.

Distances to water systems vary markedly, ranging from 0 m to 8,608.85 m. The mean distance (~2,300 m) exceeds the median (~1,500 m), indicating a right-skewed distribution. Villages below the median are typically located within 480 m of water sources, whereas villages above the mean are often more than 4,100 m away, highlighting substantial variability in water-system accessibility.

Based on these indicators, K-means clustering identified five environmental types (A–E) (Table 2). Type A (n = 66) comprises low-elevation settlements with moderate terrain complexity and relatively greater distances to water systems. Type B (n = 76) includes mid-elevation villages characterized by pronounced topographic relief and heterogeneous slope orientations. Type C (n = 50) consists of high-elevation villages with the greatest average distances to water systems. Type D (n = 26) is distinguished by substantial variability in distance to water systems, while Type E (n = 94) represents mid-elevation settlements with minimal relief, favorable slope orientations, and relatively uniform water accessibility.

**Table 2:** Distribution of villages across environmental indicator levels in five environmental types.

Environmental type	Indicator	Lowest (20%)	Lower (20%)	Middle (20%)	Upper (20%)	Highest (20%)
**A**	elevation (m)	18	18	15	10	5
	topographic relief (m)	2	10	14	23	17
	slope aspect (°)	37	26	3	0	0
	water systems distance (m)	13	13	16	16	8
**B**	elevation (m)	17	18	17	18	6
	topographic relief (m)	0	2	16	26	32
	slope aspect (°)	0	0	10	26	40
	water systems distance (m)	23	16	16	17	4
**C**	elevation (m)	0	2	6	6	36
	topographic relief (m)	8	12	15	9	6
	slope aspect (°)	5	10	13	13	9
	water systems distance (m)	2	2	3	11	32
**D**	elevation (m)	6	4	5	7	4
	topographic relief (m)	1	4	7	5	9
	slope aspect (°)	9	4	5	6	2
	water systems distance (m)	0	0	3	5	18
**E**	elevation (m)	21	21	19	20	13
	topographic relief (m)	50	31	9	4	0
	slope aspect (°)	10	26	29	17	12
	water systems distance (m)	25	31	24	13	1

Note: 1. All numerical values inside cells represent the count of ethnic minority villages; the labels “Lowest (20%)–Highest (20%)” stand for five equal quantile groups of each environmental variable, not cell percentages. 2. Measurement units: elevation, topographic relief and water systems distance are measured in meters (m); slope aspect is measured in degrees (°). 3. Five environmental types (A–E) are derived from K-means clustering based on standardized environmental variables, consistent with the gradient classification standard used in Figure 6.

At the prefectural scale (Table 3), central cities such as Guiyang and Anshun are dominated by Type E environments. Northern cities display mixed compositions of Types B and E, while eastern and southern autonomous prefectures exhibit diversified environmental structures containing all five types. Western regions are predominantly characterized by Type C environments. These patterns indicate significant regional differences in the environmental context of ethnic minority villages. Typification can capture and represent the characteristics of these differences (Fig 5).

**Table 3:** Distribution of environmental types across cities and prefectures in Guizhou Province.

City / Prefecture	Type A	Type B	Type C	Type D	Type E
**Qiandongnan Miao and Dong Autonomous Prefecture**	29	36	10	20	31
**Tongren City**	13	5	2	1	14
**Qiannan Buyi and Miao Autonomous Prefecture**	9	12	4	3	10
**Anshun City**	5	4	1	0	13
**Qianxinan Buyi and Miao Autonomous Prefecture**	4	7	6	1	10
**Guiyang City**	3	3	1	0	9
**Bijie City**	2	2	14	0	0
**Zunyi City**	1	5	3	1	5
**Liupanshui City**	0	2	9	0	2

Note: All cell values represent the count of ethnic minority villages. Types A–E refer to the five environmental clusters defined in Table 2

**Fig 5:**
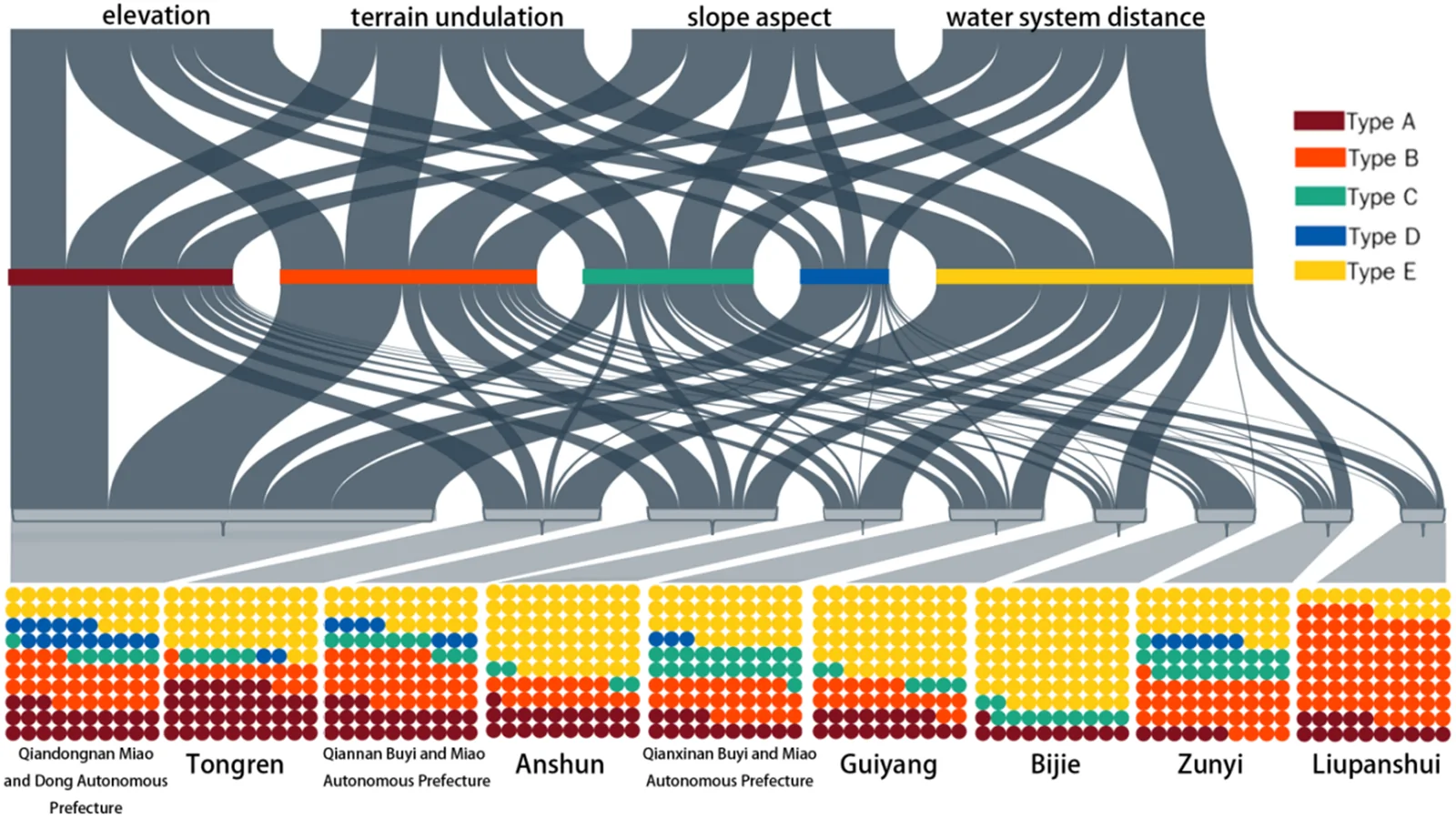
Composition of environmental types of ethnic minority villages across cities and autonomous prefectures. Environmental types were derived using K-means clustering based on standardized environmental variables.

### Recessiveness structure of Guizhou’s cultural landscape

A total of 305 ICH items were documented across the 312 ethnic minority villages. Seven villages lack registered ICH items; these villages were retained in environmental clustering and spatial mapping but excluded from recessiveness scoring and gradient analyses due to missing ICH data.

Based on the composite recessiveness index, Guizhou’s cultural landscapes are predominantly characterized by medium-level recessiveness (Table 4). These classifications relate to the combined profile of observability, carrier explicitness, and environmental association rather than inherent properties of cultural traditions themselves.

**Table 4:** Distribution of recessiveness levels, environmental types, and ICH domains across municipal administrative units.

City / Prefecture	Recessiveness levels			Environmental type					ICH domains				
	low	moderately	high	A	B	C	D	E	nature & universe	oral traditions	craftsmanship	social practices	performing arts
**Qiandongnan**	10	95	17	27	36	9	20	30	11	18	16	61	16
**Tongren**	1	32	2	13	5	2	1	14	3	6	4	18	4
**Qiannan**	3	29	6	9	12	4	3	10	3	2	1	30	2
**Anshun**	2	20	1	5	4	1	0	13	1	2	6	11	3
**Qianxinan**	4	15	9	4	7	6	1	10	2	2	2	19	3
**Guiyang**	3	11	2	3	3	1	0	9	0	0	2	13	1
**Bijie**	3	11	3	1	2	14	0	0	0	1	3	12	1
**Zunyi**	4	10	1	1	5	3	1	5	0	0	2	12	1
**Liupanshui**	2	0	9	0	1	9	0	1	0	0	1	9	1

Note: 1. All cell values represent the count of registered ICH items, not percentage values. 2. Low/moderately/high recessiveness categories are classified via Jenks natural breaks based on the composite recessiveness index. 3. Types A–E denote the five environmental clusters defined in Table 2.4. Abbreviated ICH domain names correspond to official UNESCO categories: Nature & universe = Knowledge and practices concerning nature and the universe; Oral traditions = Oral traditions and expressions; Craftsmanship = Traditional craftsmanship; Social practices = Social practices, rituals and festive events; Performing arts = Performing arts.

Western Guizhou exhibits a comparatively high proportion of high-recessiveness classifications. For example, in Liupanshui, 9 of 11 ICH items (81.8%) fall into the high-recessiveness category, whereas Bijie is dominated by medium-level classifications, accounting for approximately two-thirds of documented items.

Northern and central regions, including Guiyang, Anshun, Zunyi, and Tongren, are generally dominated by medium-level recessiveness, accounting for the majority of documented items, typically more than two-thirds and exceeding 85% in several cases such as Tongren and Anshun. Low- and high-recessiveness categories occur only marginally in most of these regions.

Eastern and southern autonomous prefectures—Qiandongnan, Qiannan, and Qianxinan—exhibit more diversified recessiveness compositions. While medium-level classifications remain dominant (approximately 74–78%), both low- and high-recessiveness categories are consistently present, resulting in more heterogeneous distributional profiles.

At the provincial scale, a west–east gradient in composite recessiveness is observable. Western regions display relatively higher proportions of high-recessiveness cases, northern and central regions show more structurally concentrated medium-level profiles, and southeastern regions demonstrate diversified compositions combining low, medium, and high categories.

### Environmental gradients and cultural landscape differentiation

To examine spatial coupling between environmental differentiation and cultural landscape recessiveness, a multidimensional gradient matrix was constructed using normalized indicators of elevation, topographic relief, slope aspect, and distance to water systems (Fig 6). Each indicator was discretized into five ordinal levels, and proportional compositions were calculated for each environmental type identified via K-means clustering.

**Fig 6:**
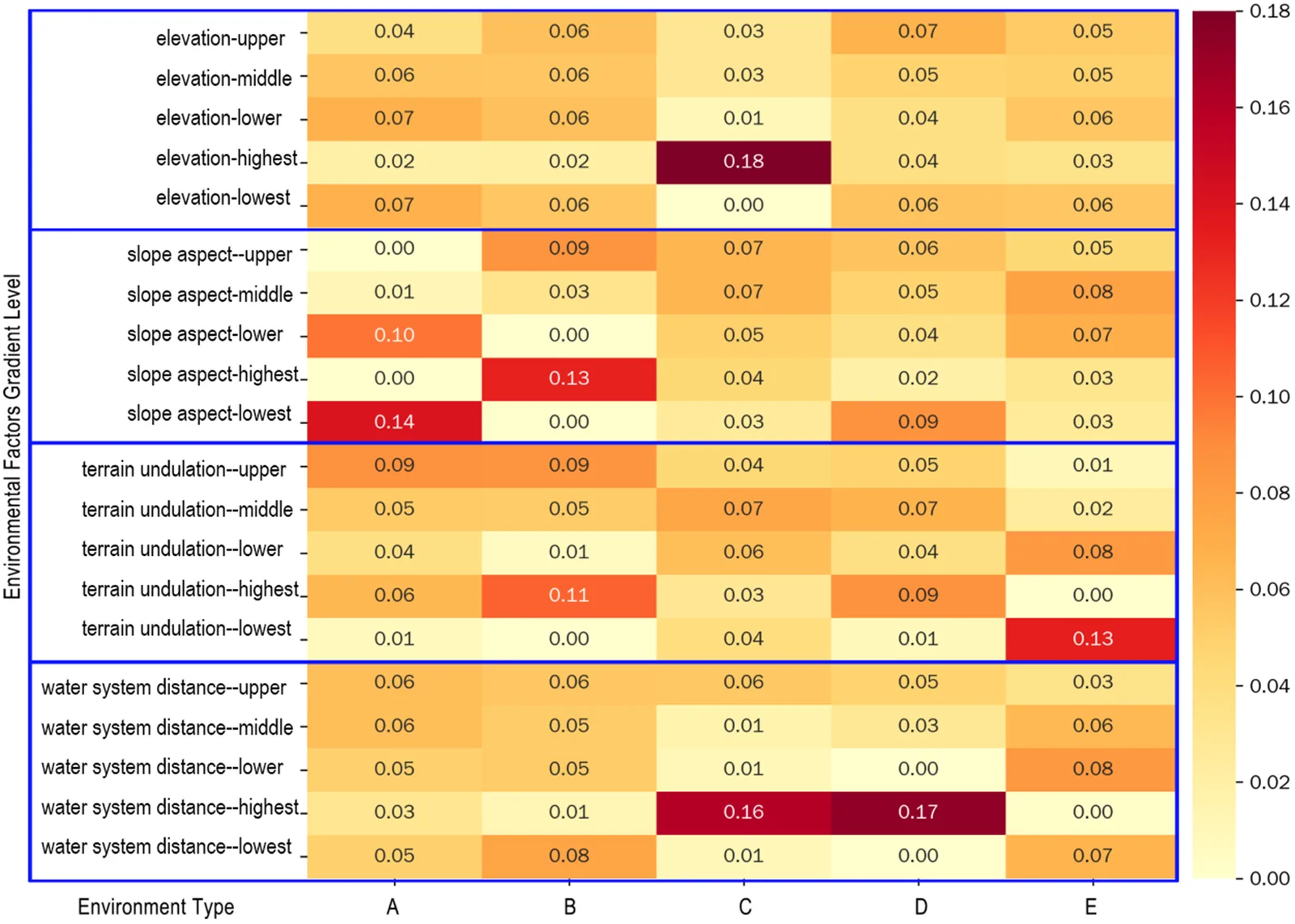
Environmental gradient distributions across five environmental types. All variables were standardized and discretized into five equal 20% quantile levels, namely Lowest, Lower, Middle, Upper, Highest, consistent with the quantile grouping criteria adopted in Table 2. Colour gradients indicate increasing environmental intensity from low (yellow colours) to high (red colours), based on standardized variables.

The resulting gradient structure reveals distinct environmental signatures across the five types. Type A is characterized by moderate values across all indicators; Type B by elevated relief and heterogeneous slope orientations; Type C by extreme elevation and greater distances to water systems; Type D by pronounced variability in distance to water systems; and Type E by relatively low relief and shorter distances to water systems.

Examination of recessiveness levels across these ecological gradients (Fig 7) reveals distinct patterns. Type A environments are dominated by moderate recessiveness. Type B features a mix of low and moderate levels. Type C hosts the highest share of high-recessiveness heritage. Type D exhibits a polarized distribution, whereas Type E shows relatively low recessiveness overall. Spatial associations between ecological settings and village-level recessiveness profiles are observed.

**Fig 7:**
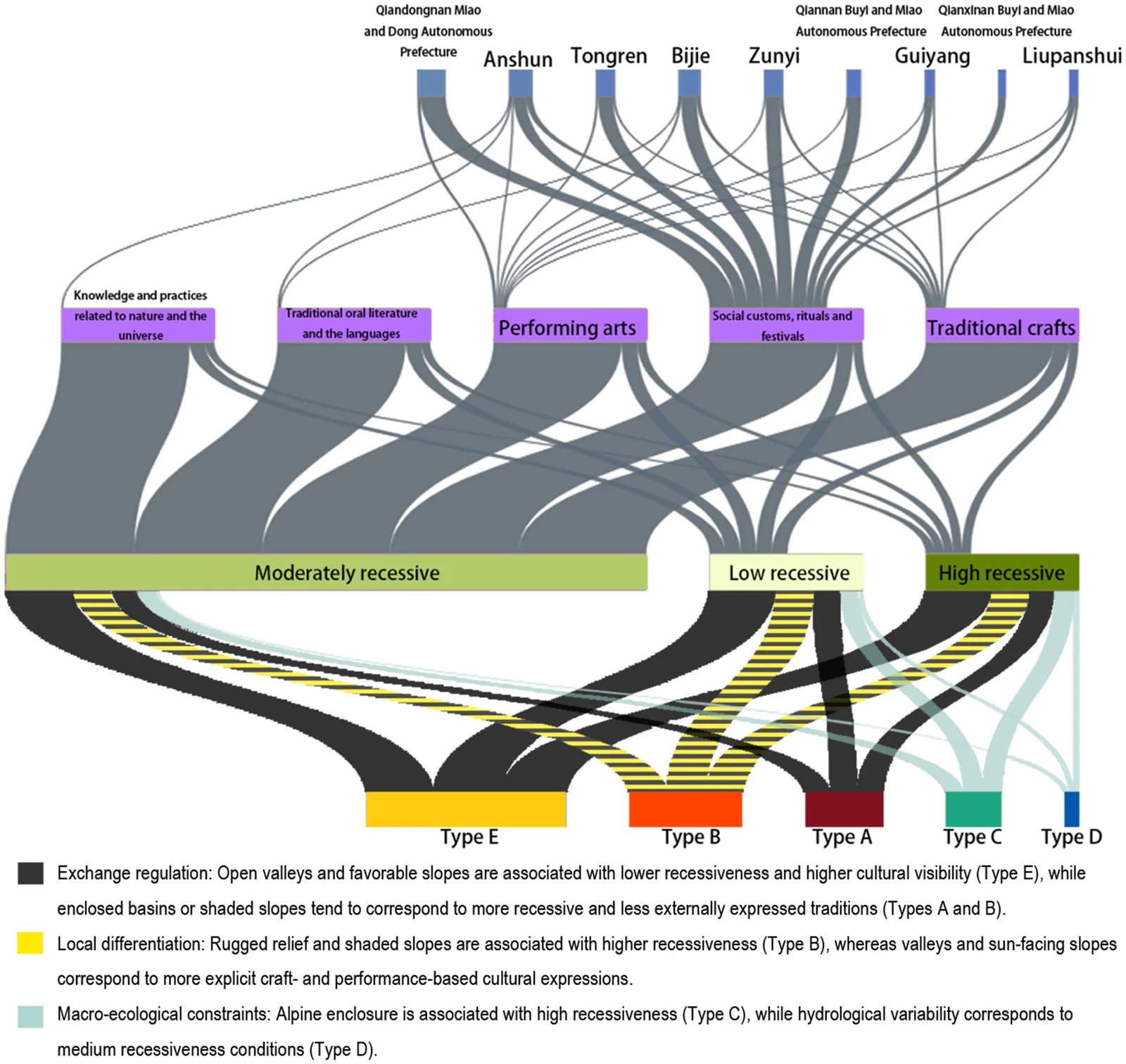
Spatial associations between environmental types, content types, and ICH recessiveness levels across municipal administrative units. Environmental types were derived using K-means clustering, and ICH recessiveness was classified using Jenks natural breaks. All variables were standardized prior to analysis.

Multiple random initializations of K-means clustering and standardized normalization were performed prior to gradient construction. The stability of classification outcomes under repeated clustering runs suggests that the differentiation observed across environmental types reflects structural regularities in the dataset rather than artifacts of algorithmic initialization.

## Discussion

The ERC framework makes three key methodological contributions to regional ICH analysis. First, it extends beyond value-oriented tangible heritage evaluation by operationalizing recessiveness as a comparative framework for context-dependent ICH. Second, it integrates environmental regimes into comparative heritage analysis rather than treating the environment as a static contextual backdrop. Third, it integrates environmental clustering, recessiveness scoring, and gradient analysis into a reproducible workflow for quantifying relationships between environment and heritage across mountainous regions, providing a standardized method for comparative heritage analysis.

### Environmental structuring of heritage landscape carriers

The spatial arrangement of heritage landscape carriers in Guizhou, namely ethnic minority villages and their affiliated ICH, indicates consistent associations between environmental heterogeneity and spatial patterns of heritage. Localized clustering coexists with province-wide dispersion, suggesting that heritage carriers are distributed across diverse ecological settings rather than aggregating within a single dominant environmental regime.

The five environmental regimes (Types A–E) represent recurrent configurations of elevation, terrain complexity, slope aspect, and distance to water systems. These regimes delineate distinct ecological settings within which settlement continuity, terrain accessibility, and environmental exposure vary systematically, and such variation corresponds to measurable disparities in composite recessiveness profiles.

### Environmental differentiation and heritage configuration

Ecological variation is associated with distinct recessiveness distributions across Guizhou. The five ecological types align with distinct aggregate distributions of low, moderate, and high recessiveness categories.

Types A and B are characterized by intermediate environmental diversity and are associated with mixed recessiveness compositions dominated by medium-level classifications. Type C environments, which are defined by higher elevation and greater distance to water systems, display comparatively higher proportions of high-recessiveness classifications. Type E environments, which are characterized by lower relief and greater accessibility, exhibit lower overall recessiveness levels. Type D environments show internally differentiated compositions that reflect the spatial coexistence of contrasting water-access conditions.

These patterns suggest associations between environmental differentiation and recessiveness classification under the ERC framework, a composite analytical metric derived from observability, carrier explicitness, and environmental association.

### Environmental gradients and heritage spatial patterns

Across environmental regimes, recessiveness classifications align with interpretable gradient-based variation. High‑elevation settings with large distance to water systems (Type C) correspond to higher concentrations of high-recessiveness heritage, while low‑relief, accessible landscapes (Type E) show lower recessiveness levels. The observed gradients indicate recurring spatial differences in heritage distribution across village environments.

Recessiveness is an analytical construct that captures the balance between context-dependent and observable heritage forms. These patterns reflect statistical association, rather than environmental determinism.

### Implications for heritage science

The ERC system translates qualitative dimensions, including observability, carrier explicitness, and environmental association, into a structured scoring model that enables spatial comparison across heterogeneous ecological regimes.

The operationalization provides a consistent basis for identifying heritage differences across environmental contexts. Reliability analyses confirm stable internal consistency, and scoring by a 10‑member expert panel reduces individual bias and enhances robustness. Furthermore, clustering stability across multiple initializations and consistent gradient patterns support the robustness of environment–heritage associations. These advances support the ERC framework as a quantitative approach moving beyond descriptive case studies toward standardized environment–ICH analysis.

Integrating gradient analysis with patterned heritage classification extends ecological concepts to intangible cultural landscape research. The approach enables heritage configuration to be examined via reproducible spatial metrics.

### Practical implications for Heritage Governance in mountainous multi-ethnic regions

The environmental–ICH coupling patterns extracted by the ERC framework provide quantitative spatial evidence for interpreting distributions of mountain cultural landscapes. By integrating environmental classification with a composite recessiveness metric, the framework addresses a key methodological gap in spatially differentiated heritage decision-making. ERC can be interpreted as a replicable decision-support framework that integrates environmental typology, recessiveness grading, and spatial gradient analysis. Across heterogeneous mountain terrain, High-elevation and isolated settings tend to correspond to higher proportions of highly recessive intangible heritage, whereas low-relief and accessible areas are associated with relatively more visible cultural forms. Transitional zones are associated with differentiated governance strategies. The composite recessiveness index further informs conservation prioritization by linking ecological context to cultural visibility and supports more efficient allocation of limited heritage resources.

The ERC workflow is transferable to other multi-ethnic mountainous regions with similar environmental heterogeneity, with Guizhou serving as the empirical case study. All observed relationships reflect spatial covariation rather than causal mechanisms.

### Limitations and future perspectives

Several limitations should be acknowledged. First, while supported by expert evaluation and adequate reliability, the ERC framework remains an operational construct. Classification outcomes depend on indicator definition and scoring interpretation. Future work could strengthen robustness via cross‑regional validation, Delphi refinement, or longitudinal testing. Four core environmental indicators were selected: elevation, relief, slope aspect, and distance to water systems, as these variables capture major topographic and water-access conditions influencing settlement distribution, accessibility, and environmental exposure across Guizhou’s mountainous landscapes. Bioclimatic and soil variables may also influence heritage distribution, but they generally operate at finer ecological scales and were not incorporated into the present regional classification framework. Multi‑scale bioclimatic and edaphic data could refine environmental classification in future studies.

Second, the analysis focuses on environmental patterns and does not incorporate socio-economic or institutional factors such as migration, market integration, tourism development, or governance. These factors likely interact with environmental gradients in shaping heritage configuration. Integrating environmental and social variables through multilevel or temporal analysis could deepen explanatory understanding.

Finally, the framework identifies reproducible statistical associations but does not establish causal mechanisms. Its primary contribution lies in demonstrating structured spatial links between ecological heterogeneity and heritage configuration. Continued studies across mountain regions are needed to evaluate the generalizability of the environment–heritage coupling perspective.

## Conclusion

This study demonstrates that the spatial distribution and recessiveness patterns of ICH across Guizhou correspond to five environmental regimes delineated based on elevation, terrain complexity, slope aspect, and distance to water systems.

The results reveal distinct spatial regularities: high-elevation terrains with large distance to water systems are associated with higher proportions of high-recessiveness heritage, whereas low-relief and highly accessible landscapes are associated with predominantly low-recessiveness ICH. Intermediate environmental types display mixed recessiveness compositions. Such spatial disparities suggest that ecological differentiation is associated with systematic differences in recessiveness composition, with recessiveness serving as a standardized analytical metric for quantifying varying degrees of heritage embeddedness. Environmental heterogeneity is statistically associated with spatial divergence in heritage distribution, indicating correlation rather than causation. Beyond Guizhou, the ERC framework provides a comparative basis for examining environment-heritage relationships across complex mountain regions.

## Supporting information

S1 Table Basic environmental analysis data of 312 villages. (XLSX)

S2 Table Raw expert scoring matrix for ICH recessiveness evaluation. (PDF)
