# Supplementary material for: An environmental type-recessiveness framework for the spatial coupling of environmental heterogeneity and ICH recessiveness in Guizhou’s ethnic minority villages, China
Source: PLoS One. 2026 Oct 22;21(10):e0360100. doi: 10.1371/journal.pone.0360100 (PMC13690210; doi:10.1371/journal.pone.0360100)
Supplement: Supplementary file 1 [file pone.0360100.s002.pdf]

Sample No	Village	E1 Obs	E2 Obs	E3 Obs	E4 Obs	E5 Obs	E6 Obs	E7 Obs	E8 Obs	E9 Obs	E10 Obs	E1 Car	E2 Car	E3 Car	E4 Car	E5 Car	E6 Car	E7 Car	E8 Car	E9 Car	E10 Car	E1 Env	E2 Env	E3 Env	E4 Env	E5 Env	E6 Env	E7 Env	E8 Env	E9 Env	E10 Env
1	Pianpo Village	2	2	2	2	2	2	1	2	2	1	2	3	2	2	2	2	2	2	2	2	2	3	2	2	3	1	2	3	2	2
2	Xiayuan Village	1	2	2	2	2	2	2	2	2	2	2	2	2	3	2	3	2	2	2	1	2	1	2	2	3	2	3	2	2	3
3	Wanggang Village	2	3	2	2	2	3	1	2	2	2	2	3	2	3	2	2	2	3	2	1	2	2	2	3	2	2	3	2	1	2
4	Longjing Village	2	2	2	2	2	2	1	2	2	2	2	2	2	2	3	2	2	2	2	2	2	2	2	2	2	2	2	2	2	2
5	Dachong Village, Hu	2	2	2	2	3	2	3	2	2	2	2	3	1	3	3	2	2	1	3	2	2	2	3	2	2	2	2	3	2	2
6	Longguang Village	2	2	3	2	2	2	2	2	2	2	2	2	2	2	2	2	2	2	2	2	2	1	2	3	2	3	2	2	2	2
7	Malangdie Village	1	2	2	2	2	2	2	2	2	1	2	2	1	3	2	2	1	2	2	2	2	2	2	2	2	2	1	2	2	2
8	Minzu Village	2	2	2	2	2	1	2	2	2	2	2	2	2	2	2	2	2	2	2	2	2	2	2	2	2	2	2	2	1	2
9	Hongxin Village	2	2	2	2	2	2	2	2	2	2	2	2	3	1	2	3	3	2	2	2	2	2	2	2	2	3	2	2	2	2
10	Tuhe Village	3	3	3	3	3	3	3	2	2	3	3	3	3	2	2	3	3	2	2	2	3	3	3	2	2	2	2	4	2	3
11	Sangmuba Village	2	1	2	2	2	2	3	2	2	2	2	2	2	2	2	2	2	3	2	2	3	2	2	2	2	2	2	2	3	2
12	Longtan Village	2	2	2	2	2	2	1	1	2	1	2	3	2	2	2	3	3	1	2	2	2	2	2	3	2	2	3	3	2	2
13	Shangxing Village	1	1	2	2	1	2	1	2	1	1	2	1	1	1	2	2	1	1	1	1	2	2	2	1	1	1	2	1	2	1
14	Mucheng Village	2	2	3	1	3	2	2	2	2	2	3	2	2	1	2	2	2	2	2	3	2	2	3	2	2	2	2	2	2	2
15	Gaodang Village	2	1	2	2	2	3	2	2	2	2	2	2	2	2	2	2	1	2	2	3	2	1	2	1	2	3	1	2	2	2
16	Shitouzhai Village	2	1	2	2	2	2	3	2	2	2	3	2	3	2	3	2	3	2	2	2	2	2	2	2	3	2	2	2	2	2
17	Huashishao Village	4	4	3	2	3	2	3	3	2	3	3	3	4	4	3	3	3	3	3	3	3	3	2	2	2	3	3	3	2	3
18	Fuyuan Village	3	2	2	2	2	2	1	2	3	3	2	2	1	2	3	2	3	2	2	2	2	2	2	3	2	2	3	2	3	3
19	Jiucaiping Village	1	1	1	1	2	1	1	2	2	1	2	1	2	1	1	2	1	2	2	1	2	1	2	1	2	2	2	1	2	1
20	Bandi Village	1	1	2	1	2	1	1	2	2	1	2	2	2	1	2	1	1	1	2	1	2	2	2	1	2	1	1	2	1	1
21	Qingnianhu Village	2	2	2	2	1	1	2	2	2	2	2	2	2	3	2	2	2	2	2	2	2	2	2	2	2	2	2	2	2	2
22	Fanjingshan Village	2	1	2	2	2	2	2	3	2	2	2	3	2	2	2	2	2	2	3	2	2	2	3	1	2	3	2	2	2	2
23	Yunshe Village	2	2	2	2	2	2	2	2	2	2	2	2	2	2	2	2	2	2	2	2	1	1	2	2	2	1	2	2	1	2
24	Yaoshang Village	2	2	2	2	3	2	2	2	2	3	2	2	2	2	3	2	2	2	3	2	2	3	2	2	2	2	3	2	2	2
25	Loushang Village	2	2	2	2	2	2	2	2	2	2	2	1	2	1	2	2	2	2	2	2	2	1	2	3	2	2	2	1	2	2
26	Yuanyanghu Village	3	2	2	2	2	2	4	2	2	3	2	3	3	3	2	2	3	2	3	2	2	3	2	4	3	2	3	3	4	4
27	Tuanlong Village	2	2	2	2	3	2	2	2	2	2	2	2	2	3	2	2	2	2	2	2	2	2	2	1	2	2	2	3	2	2
28	Nanzhuang Village	3	2	2	2	2	2	2	2	2	2	2	2	3	1	2	2	2	2	2	3	2	2	2	2	2	2	2	2	2	2
29	Haocai Village	3	3	2	2	3	4	3	3	2	3	3	3	3	2	3	2	3	3	3	3	2	3	2	3	3	3	2	3	3	3
30	Dawan Village	2	2	3	2	2	2	2	2	2	2	1	1	2	2	1	2	2	2	2	2	2	2	2	2	2	2	2	2	2	2
31	Xiangshuidong Villa	2	2	2	2	2	2	2	2	2	2	2	2	2	2	2	2	2	2	2	2	2	3	2	2	3	3	2	2	2	2
32	Wuli Zhai	2	2	1	3	2	2	2	2	2	2	2	2	2	2	2	2	2	2	2	2	1	2	2	2	3	1	2	1	2	3
33	Sanmentang Village	2	3	2	2	2	2	2	2	2	1	2	2	2	2	2	2	2	2	2	3	2	3	2	2	2	2	2	2	2	1
34	Longlisuo Village	2	2	2	1	2	1	2	1	2	2	2	2	2	2	2	2	2	2	2	2	2	2	1	1	2	3	3	2	2	1
35	Tunzhou Village	2	2	2	3	2	3	3	3	2	2	3	2	2	2	2	2	3	2	2	2	2	1	2	2	2	2	2	2	2	2
36	Jiaogong Village	2	2	2	2	2	3	2	2	3	2	2	2	2	2	2	2	3	2	2	2	2	3	2	3	3	2	2	1	2	2
37	Zhaoxing Dong Zhai	3	3	2	3	2	3	2	3	2	2	3	3	3	3	2	3	4	3	3	3	2	4	2	3	3	3	2	3	2	2
38	Sizhai Village	1	2	1	2	2	1	2	2	2	2	2	2	2	2	2	2	2	2	2	2	2	3	2	2	3	1	2	3	2	2
39	Tongguan Village	2	2	3	1	3	3	2	2	2	1	2	2	2	2	2	2	2	2	2	2	2	2	2	2	2	1	2	2	2	2
40	Xiaodanjiang Villa	3	4	3	3	3	3	3	2	2	3	3	3	3	3	2	2	3	2	3	2	2	2	3	2	3	4	3	3	3	3
41	Xijiang Village	1	1	2	2	2	2	1	1	2	1	1	1	1	1	2	2	1	2	1	1	2	1	2	1	1	2	2	1	1	1
42	Fuxing Village	3	2	2	2	3	2	2	2	2	2	2	1	2	3	2	2	3	2	2	2	1	2	2	1	3	1	2	2	2	2
43	Liubao Village	2	3	3	3	2	4	4	2	2	3	3	2	4	2	2	2	3	3	2	3	3	3	2	3	3	2	3	3	3	3
44	Kala Village	3	3	3	2	2	3	3	3	2	3	2	3	3	2	2	2	3	3	3	2	3	3	2	4	2	3	2	3	4	3
45	Shiqiao Village	2	2	2	2	2	2	3	3	2	2	2	2	2	2	2	2	1	2	2	2	2	2	3	3	2	2	2	3	2	2
46	Bagu Village, Pojia	2	2	2	2	2	3	2	3	2	2	2	2	2	3	2	2	1	2	2	1	2	1	2	2	2	3	2	2	2	1
47	Huangsi Village	1	3	2	2	2	2	1	2	2	2	2	2	2	1	1	2	3	2	2	1	2	2	2	2	2	1	2	3	2	3
48	Lapian Village	1	3	2	2	3	3	2	2	2	2	2	2	2	2	2	2	2	2	2	2	3	3	2	2	2	2	2	3	2	2
49	Yin Zhai Village	3	3	3	2	3	3	3	2	2	3	2	3	3	3	3	2	3	3	3	3	3	3	3	3	3	4	2	3	3	4
50	Wengqi Village	2	2	2	1	2	2	2	2	2	1	2	2	1	2	2	2	2	1	2	1	2	2	2	2	2	3	2	2	2	2
51	Changhe Village	1	1	2	2	1	2	1	2	1	2	2	1	1	2	1	1	1	2	1	1	2	1	2	1	2	1	2	2	1	2
52	Haohua Hong Village	2	2	2	2	1	2	2	3	2	2	2	1	2	1	2	2	3	1	2	2	2	3	2	2	2	2	2	2	2	2
53	Jiulong Village	3	3	3	2	3	3	3	2	2	3	2	3	3	3	3	2	3	3	3	4	3	4	3	3	2	4	3	2	3	3
54	Guguan Village	3	2	2	2	2	2	1	2	2	3	2	2	3	2	2	2	1	2	3	3	2	3	2	2	3	2	2	1	2	1
55	Nahui Village	1	2	1	2	2	2	3	2	2	1	2	2	3	1	2	2	2	2	2	2	2	1	1	2	2	2	2	2	2	1
56	Louna Village	2	2	2	2	2	3	2	2	2	2	2	2	1	2	3	2	2	1	2	2	2	2	2	1	2	2	2	2	2	3
57	Minzu Village	3	2	2	2	3	3	2	2	3	2	3	3	3	2	1	3	1	3	2	3	2	2	2	3	2	3	3	2	2	3
58	Liyuba Village	2	2	2	2	3	2	2	2	2	2	2	2	2	2	2	2	2	3	2	2	2	1	2	2	2	1	2	3	3	2
59	Luchuying Village	1	1	2	2	2	3	1	2	2	2	2	2	2	2	2	2	2	2	2	1	3	2	2	2	2	2	2	2	2	2
60	Zheguyuan Village	1	1	1	2	2	1	1	2	2	1	1	2	1	1	1	2	1	2	1	1	1	1	2	1	2	1	2	1	1	2
61	Nakong Village	3	3	2	2	3	3	3	3	2	2	3	2	3	3	2	2	3	2	2	3	3	4	3	3	3	3	3	3	3	2
62	Dadang Village	2	2	2	2	3	1	3	2	2	3	2	2	2	2	2	2	2	2	2	2	2	2	1	2	2	1	2	2	2	2
63	Zhenshan Village	2	2	2	2	2	2	2	2	2	2	2	2	2	2	2	2	2	2	2	2	2	2	2	1	2	2	2	2	2	2
64	Maiweng Zhai	3	3	2	2	2	3	3	3	3	3	3	4	3	4	3	3	3	3	2	3	3	4	3	3	2	3	2	3	2	3
65	Huanglian Village	2	2	2	2	2	3	2	2	2	2	2	2	2	2	2	2	2	2	2	2	2	2	2	2	2	2	2	2	2	3
66	Longshang Village	2	3	2	2	2	2	2	2	2	2	1	1	2	1	2	2	2	2	2	3	2	2	2	2	2	2	2	2	2	2
67	Matou Village	3	3	2	2	3	3	3	2	2	2	2	3	2	4	2	3	3	2	3	4	2	3	2	3	4	3	2	3	3	3
68	Chuandong Village,	2	1	1	2	2	2	1	2	2	1	2	1	1	1	1	2	1	2	1	1	2	1	2	1	2	1	2	2	2	1
69	Miao Zhai Village	3	2	2	3	2	2	3	3	2	3	2	3	3	3	2	3	3	2	3	3	2	2	2	2	2	2	3	3	2	3
70	Yanyingshan Village	2	2	2	2	1	1	2	2	3	2	2	2	3	3	2	3	2	1	3	1	2	2	2	2	1	2	1	2	1	1
71	Longtai Village	2	2	2	2	2	2	2	2	2	1	2	3	2	2	2	2	1	2	2	2	3	2	3	2	2	3	2	2	3	3
72	Datang Village	3	3	3	3	2	1	2	2	2	2	3	2	2	2	2	2	2	3	2	2	2	2	2	2	2	1	2	2	2	2
73	Diguan Village, Pi	1	2	2	2	1	2	2	2	2	2	2	2	2	3	2	2	2	2	2	2	3	2	2	2	2	3	3	2	2	1
74	Taofu Village	1	1	2	3	2	2	3	2	2	2	2	2	3	3	2	3	2	2	2	2	2	2	2	2	2	2	2	3	2	2
75	Miaoba Village	1	1	2	1	2	2	2	2	2	2	2	2	2	2	2	2	2	2	2	2	2	2	1	2	2	2	2	2	2	1
76	Huashan Village, Fe	3	2	2	2	2	2	2	2	2	2	2	2	2	2	2	2	2	2	2	1	2	1	2	2	2	2	2	3	2	2
77	Daba Village	3	3	2	2	2	2	2	2	3	1	2	2	2	2	2	2	3	3	2	2	2	1	2	2	2	3	3	3	3	2
78	Niujiao Village	2	3	2	2	2	2	2	2	2	2	2	2	1	2	2	1	2	2	2	3	1	2	2	2	2	2	2	2	2	2
79	Gaoxing Village	3	2	2	2	2	1	2	2	2	3	2	2	2	2	2	2	2	2	2	2	2	2	2	3	2	1	2	1	2	2
80	Buna Village	3	3	2	2	2	3	3	3	2	3	3	3	2	4	3	2	3	2	2	3	2	3	3	3	2	3	3	3	3	3

81	Xinfa Village	1	1	2	2	1	2	1	2	2	1	1	1	2	1	2	2	1	2	2	1	1	2	1	1	2	1	1	2	2	1
82	Haiping Village	2	2	3	2	2	2	2	3	2	2	2	1	2	3	2	2	2	2	2	1	2	1	2	2	3	2	2	2	2	2
83	Namu Village, Guan	2	2	2	3	2	2	2	2	2	2	2	2	2	2	3	3	2	2	2	2	2	2	2	3	2	2	2	2	2	2
84	Heqiao Village	1	3	2	2	2	2	2	2	2	2	2	1	2	3	2	2	2	3	3	2	2	2	2	3	2	2	2	2	2	2
85	Longqing Village	3	3	3	2	2	2	3	2	2	4	3	3	3	3	3	3	3	3	3	4	2	2	3	3	3	3	2	2	3	3
86	Pingtian Village	2	3	3	2	2	3	2	2	2	2	2	2	2	2	2	2	1	2	2	2	2	3	2	1	2	2	2	1	2	2
87	Dazhai Village	1	2	2	2	1	1	2	1	1	1	2	1	1	1	2	2	1	1	2	1	2	1	2	1	2	1	1	1	1	2
88	Xiaohewan Village	3	2	2	2	2	2	2	2	3	2	2	2	1	2	2	2	2	2	2	2	2	2	2	2	2	2	2	2	2	2
89	Xingjiang Village	2	3	2	2	3	1	1	2	2	2	2	3	2	2	2	3	2	2	2	2	2	2	2	2	3	2	2	2	3	2
90	Longcai Village	1	1	2	2	1	2	1	2	2	1	2	2	1	1	1	2	2	1	2	1	2	1	1	1	1	1	2	1	2	1
91	Yueliangwan Villag	3	2	2	2	2	2	2	2	2	2	2	2	1	2	2	2	3	2	2	2	2	2	2	3	2	1	3	1	1	2
92	Bazhai Village	2	2	2	2	2	2	2	2	3	2	2	2	2	2	2	2	2	3	2	2	2	2	2	2	2	2	2	1	2	3
93	Pingzhai Village, S	2	2	2	2	3	2	2	2	3	2	2	2	2	2	2	2	1	2	2	1	2	3	2	2	2	2	2	3	2	2
94	Longtan Village	1	1	2	1	1	1	2	2	2	1	2	1	1	1	2	2	1	1	1	2	2	1	1	1	1	1	1	1	1	2
95	Zhonghuashan Villa	2	2	2	2	2	2	2	3	2	2	2	2	2	2	2	2	2	2	2	2	2	2	1	2	2	2	2	2	2	2
96	Zhaibao Village	3	2	3	2	2	2	2	2	2	1	2	2	2	2	2	2	2	2	2	2	3	2	2	2	2	2	2	1	2	2
97	Yundu Village, Qian	2	2	2	2	2	2	2	1	2	2	2	1	2	3	2	2	2	2	2	2	2	3	2	3	3	2	2	2	2	1
98	Kuaichang Village,	1	2	3	2	3	2	3	2	3	2	2	3	2	1	2	2	2	2	2	1	2	2	3	2	2	2	2	2	2	2
99	Yejiping Village	2	2	2	2	2	2	2	2	2	2	2	3	2	2	2	2	2	2	2	2	2	2	2	2	2	2	2	2	2	2
100	Shenxianmiao Villa	2	2	3	2	2	2	2	3	2	3	2	2	2	2	2	2	2	2	2	2	2	1	3	3	2	2	3	2	2	3
101	Hexi Village	2	1	2	1	2	2	2	2	2	2	2	2	2	2	2	2	2	2	2	2	1	2	2	2	2	2	3	2	3	2
102	Exi Village	2	2	2	2	2	2	2	2	2	2	2	2	2	2	2	2	2	2	2	2	2	3	2	1	2	3	2	2	2	2
103	Furong Village	2	3	2	2	2	2	2	2	2	2	2	2	2	2	2	2	1	2	1	2	2	2	2	2	2	2	2	2	2	1
104	Yanziyan Village	1	2	2	1	2	1	1	2	2	1	2	1	1	1	1	2	1	1	1	1	2	1	2	2	2	1	2	1	2	2
105	Hexi Village, Ganch	3	2	2	2	2	1	3	3	2	2	2	3	2	2	2	2	2	2	2	2	3	2	2	2	3	3	2	1	2	2
106	Aihong Village	1	3	2	2	2	2	2	2	2	2	1	2	3	2	2	2	1	2	2	2	2	1	2	2	2	2	2	3	3	2
107	Xinkai Village	2	2	2	3	1	2	2	2	2	1	2	2	1	2	2	2	3	2	2	1	1	3	2	2	2	2	2	2	3	2
108	Muzhai Village	2	2	2	2	2	2	2	2	2	2	2	3	2	2	2	2	2	3	2	3	3	2	2	2	2	1	2	2	2	3
109	Dianzi Village	2	2	3	2	2	2	2	2	2	2	2	2	2	1	2	2	2	1	2	2	2	2	2	1	2	2	2	1	2	2
110	Dazhai Village	2	2	3	2	2	2	2	2	2	3	2	2	2	2	2	2	3	3	2	2	2	2	2	3	3	2	2	2	2	2
111	Xiuzhong Community	3	3	3	2	3	3	3	3	2	3	3	3	3	3	2	2	3	3	2	3	3	3	3	3	2	4	3	3	2	3
112	Zhongying Village	2	2	2	2	2	2	1	2	2	3	2	2	1	2	2	3	3	2	2	3	2	3	1	1	2	2	2	1	2	2
113	Yingfeng Village	3	3	3	3	2	3	3	3	2	3	2	2	3	3	2	3	3	2	2	3	2	3	2	3	3	3	3	3	3	3
114	Mianxi Village, Mia	1	2	2	3	2	3	3	2	2	2	2	2	1	2	2	2	2	2	2	2	2	2	2	3	2	3	3	2	2	2
115	Yangduo Village, Ya	2	1	2	2	1	1	2	2	2	2	2	3	1	1	2	2	2	2	2	2	2	3	2	3	2	2	2	1	2	2
116	Langli Village, Lan	2	2	2	2	2	2	1	2	2	2	2	2	2	2	2	3	2	2	2	2	2	2	2	2	2	2	2	2	2	2
117	Nanhua Village, Nan	2	2	2	2	1	1	3	2	2	2	2	2	2	1	2	2	2	2	2	2	2	3	2	2	2	1	3	2	2	2
118	Yanzhai Village, Ya	2	2	2	2	2	2	2	2	2	2	2	1	2	2	3	2	2	1	2	3	2	2	2	2	2	2	2	2	2	2
119	Hongxi Village, Hon	2	3	3	3	2	3	3	3	2	3	3	3	3	4	2	3	3	3	3	3	3	3	3	3	3	2	3	4	2	4
120	Yingpan Village, Yi	2	2	2	2	2	2	2	2	2	2	2	3	3	2	3	3	2	2	2	2	2	1	2	2	2	2	1	1	2	2
121	Duijiang Village, Y	2	2	3	2	2	2	2	2	2	3	2	3	1	3	2	3	2	2	2	2	2	2	2	2	2	3	2	2	2	1
122	Guanzhuang Village,	1	2	1	2	2	1	2	2	2	1	2	1	1	2	2	1	1	2	2	1	2	2	1	2	2	1	2	1	2	1
123	Baiyangping Village	2	1	1	2	1	1	1	1	2	1	2	1	1	1	2	2	1	1	1	1	2	2	2	1	1	1	2	1	1	1
124	Baojing Village, Ba	3	4	3	2	2	3	3	3	3	3	3	3	2	3	3	3	4	2	3	3	2	3	2	3	3	3	3	2	3	2
125	Shanping Village	2	2	2	2	2	3	1	2	2	1	3	2	2	2	2	2	3	3	2	3	2	3	2	2	2	3	3	2	3	2
126	Shaping Village	1	2	2	3	2	2	3	2	2	2	2	2	2	2	2	2	2	2	2	2	2	2	2	2	2	2	2	2	2	2
127	Yuntai Village	4	2	2	2	2	2	3	2	3	2	2	2	3	4	3	2	3	3	2	2	2	2	3	3	2	3	3	3	2	3
128	Gaobei Village	2	2	2	2	2	2	1	3	1	1	2	3	2	2	2	2	2	3	2	3	2	1	2	2	2	3	2	2	2	1
129	Huanggu Village	2	2	2	2	2	3	2	2	2	2	2	2	1	3	2	2	2	2	2	2	3	3	3	2	2	3	2	2	2	2
130	Pingzhai Village	2	2	2	2	2	2	2	2	2	2	2	1	3	2	2	2	2	2	2	1	3	2	3	2	2	2	2	3	2	2
131	Longtang Village	1	2	2	1	2	1	2	2	2	2	2	3	1	3	2	2	2	3	3	2	2	2	2	2	2	2	2	2	2	2
132	Tonggu Village	2	3	2	2	2	1	2	2	2	2	2	2	2	2	2	3	2	1	2	2	2	2	2	2	2	1	2	2	2	2
133	Zhaitou Village	2	3	2	2	2	2	3	2	2	1	2	2	2	1	2	2	2	2	2	3	2	2	2	1	2	3	3	2	2	2
134	Yayuan Village	2	2	2	2	2	2	2	2	2	1	2	2	2	3	2	2	2	2	2	2	2	2	2	2	2	3	2	2	2	2
135	Yangliu Village	2	1	2	2	2	2	2	2	2	2	2	2	3	3	2	2	1	2	2	2	2	2	2	2	3	3	2	1	2	2
136	Baotang Village	3	3	2	3	2	3	3	2	2	3	2	3	3	2	2	2	3	3	3	3	3	3	2	3	2	2	3	4	3	3
137	Leitun Village	2	2	2	2	2	2	2	2	2	2	2	3	2	2	2	2	2	2	2	3	2	3	2	2	2	1	2	2	2	2
138	Yaobai Village	2	2	2	2	2	2	3	2	2	2	2	1	2	1	2	1	2	2	2	2	2	2	2	2	2	2	2	2	2	2
139	Wendou Village	2	2	2	2	2	3	2	2	2	2	2	3	2	2	3	2	2	2	3	3	2	1	2	2	2	2	2	2	2	2
140	Ping’ ao Village	2	2	2	2	2	2	2	2	1	1	2	1	2	1	2	1	2	2	2	1	3	3	2	2	3	2	3	2	3	3
141	Maoping Village	2	2	2	2	2	2	2	2	2	3	2	1	2	2	2	2	2	2	2	2	2	2	2	3	2	2	2	2	3	2
142	Angying Village	2	2	2	3	2	2	2	2	2	2	2	1	2	2	2	2	3	2	2	1	2	2	2	2	2	1	2	2	2	3
143	Fanzhao Village	2	1	2	1	1	1	1	2	2	1	2	1	2	1	2	1	2	1	2	1	1	1	1	1	2	1	1	1	1	1
144	Jiyou Village	2	2	2	2	2	1	3	2	2	1	2	2	2	3	2	2	2	2	2	2	1	2	2	2	2	2	2	2	2	2
145	Hongyang Village	2	2	2	2	2	2	2	2	2	3	2	1	2	1	2	2	2	2	1	2	2	3	2	1	3	2	2	3	2	2
146	Fanpai Village	2	2	2	2	2	2	3	2	2	2	2	3	2	2	2	2	2	2	1	1	2	3	3	2	2	2	2	1	2	1
147	Changtan Village	1	1	1	2	2	1	2	2	1	2	2	2	1	2	2	2	2	2	2	2	2	2	2	2	1	2	2	1	2	2
148	Jiaowang Village	2	2	2	2	1	2	2	2	2	1	2	2	2	2	3	2	2	2	2	2	1	1	2	1	2	2	2	3	2	2
149	Dimen Village	3	2	2	2	2	2	1	2	2	3	2	2	2	2	2	2	2	2	2	2	2	2	2	2	2	2	2	2	2	2
150	Huanggang Village	2	2	2	2	2	1	2	2	2	3	2	2	1	2	3	2	2	2	2	2	1	2	2	2	2	2	3	2	2	1
151	Gunzhen Village	1	3	2	2	1	1	2	2	3	2	2	3	2	2	2	2	2	2	2	2	3	2	2	2	2	3	2	1	2	2
152	Shudong Village	1	1	1	2	2	1	2	1	1	1	2	2	1	1	2	2	2	2	2	1	1	1	1	1	2	1	2	1	1	1
153	Zhongluo Village	2	2	3	2	2	2	3	2	3	2	2	2	1	2	2	2	2	2	2	3	2	2	3	2	2	2	2	2	2	1
154	Tang’ an Village	2	2	2	2	2	3	1	2	2	1	2	2	2	3	2	2	2	2	3	2	2	2	2	2	2	2	2	2	2	2
155	Dali Village	1	2	2	2	2	2	3	2	2	2	2	2	1	2	1	1	2	3	2	1	2	1	2	3	2	2	2	2	2	2
156	Zaidang Village	2	2	2	2	1	2	1	2	2	2	2	1	2	2	2	3	3	2	2	2	1	1	1	2	1	1	2	2	2	2
157	Qiuka Village	3	2	2	3	2	2	1	2	2	2	2	2	2	2	2	2	2	2	2	2	2	2	2	2	2	2	2	2	2	2
158	Basha Village	3	2	2	3	2	4	2	3	2	3	3	4	2	2	3	2	3	2	3	3	2	3	3	3	3	3	3	3	3	3
159	Gaowen Village	2	2	2	2	2	2	2	2	2	1	2	2	2	2	2	2	2	2	2	2	2	2	2	3	3	3	2	2	2	2
160	Baba Village	2	2	2	2	2	2	2	2	1	2	2	1	1	2	2	2	2	2	2	2	2	2	3	2	2	2	2	2	2	2
161	Xiaohuang Village	1	2	2	2	2	2	2	2	2	2	2	3	2	1	2	2	3	2	3	2	1	2	2	1	2	2	3	2	2	2

162	Gaoping Village	3	2	2	3	2	3	3	3	2	2	2	3	3	4	2	3	3	3	2	2	2	3	2	3	3	3	2	3	2	3
163	Liangwen Village	1	2	1	2	2	1	1	2	1	1	2	1	1	1	2	2	1	2	2	2	2	1	2	1	2	1	2	2	2	1
164	Jia Che Village	3	3	3	2	3	3	3	3	2	3	3	3	3	3	2	2	3	3	3	3	2	4	3	3	2	3	3	3	3	3
165	Jiaweng Village	2	2	2	2	2	2	2	2	2	1	3	1	2	2	3	2	3	2	2	3	2	2	2	1	2	2	2	3	1	1
166	Yemeng Village	2	2	2	2	2	2	2	2	2	2	2	3	2	3	2	2	2	3	3	1	2	1	2	2	2	3	2	2	2	2
167	Zhang' ao Village	2	2	2	2	1	2	2	2	2	1	3	3	3	1	2	2	2	2	2	2	2	2	2	1	2	3	2	2	1	2
168	Wudong Village	2	2	2	3	2	2	2	2	2	2	2	1	2	2	2	2	2	2	2	2	2	3	2	2	2	3	3	2	2	1
169	Getou Village	2	2	3	2	3	2	2	2	2	2	2	3	1	2	2	3	2	2	2	2	2	1	2	3	2	2	2	2	3	1
170	Shanglangde Villag	2	2	2	2	2	2	2	2	2	2	2	2	2	2	2	2	2	3	3	2	2	2	3	2	3	3	2	2	2	2
171	Yegai Village	2	2	2	2	2	2	2	2	3	2	2	2	3	2	2	2	3	3	2	3	2	2	2	2	2	2	3	2	2	2
172	Gongtong Village	2	2	2	2	2	2	2	2	2	2	2	2	2	2	2	2	2	2	2	2	2	2	2	2	2	2	2	2	2	2
173	Ganrong Village	2	2	2	2	2	2	2	2	2	2	3	1	2	1	2	2	2	2	2	3	2	2	2	3	2	2	2	2	3	3
174	Xinqiao Village	4	4	3	3	2	3	3	3	3	3	3	3	4	3	3	4	3	3	3	4	3	2	3	3	2	3	3	4	2	3
175	Douzai Village	3	2	2	2	2	1	2	2	3	2	2	2	2	2	2	2	3	2	2	2	1	1	2	1	2	2	2	2	1	2
176	Pingxiang Village	2	2	2	2	2	2	2	2	2	1	2	2	3	3	2	2	2	2	2	1	2	2	2	2	2	2	2	2	3	2
177	Paixiao Village	1	2	2	2	2	2	1	2	2	2	2	2	2	1	3	2	2	2	2	2	2	2	2	2	2	2	2	2	3	2
178	Wuxiang Village	4	3	3	3	3	3	3	2	2	3	3	3	2	4	3	2	4	3	3	3	3	3	3	2	2	3	3	2	3	4
179	Maliao Village	1	2	2	2	2	3	2	2	2	1	2	2	2	2	2	2	1	2	2	2	2	2	2	2	2	2	2	2	2	2
180	Maobiling Village	3	2	2	3	2	2	2	2	2	2	2	2	2	2	2	2	2	3	2	2	3	2	2	2	3	1	2	3	3	2
181	Qiaoluo Village	2	2	2	2	2	2	2	2	2	2	2	2	2	1	2	1	2	2	2	3	2	2	2	1	2	2	2	2	3	2
182	Maoping Village	1	3	2	2	2	2	2	2	2	3	2	2	2	2	2	2	2	2	2	3	2	1	2	1	2	2	2	2	2	2
183	Longjiang Village	3	3	2	2	3	2	3	2	3	3	3	3	4	2	2	2	3	3	2	2	3	3	3	2	2	3	3	3	2	3
184	Chengzhong Village	2	2	3	2	2	2	2	2	3	2	2	2	3	1	2	2	1	2	3	2	2	2	2	1	3	2	2	1	2	3
185	Kawu Village	2	1	2	2	2	2	3	2	2	2	2	2	2	2	2	3	2	2	2	2	3	2	2	2	2	2	2	3	2	2
186	Wengbao Village	2	1	2	2	2	1	2	2	2	2	2	3	1	3	2	2	2	2	2	2	2	2	2	2	2	2	3	2	2	2
187	Yangsong Village	3	3	2	3	3	2	3	2	2	3	2	3	3	3	3	3	3	3	3	3	2	2	3	2	3	3	3	3	3	4
188	Maniao Village	2	2	3	2	2	2	1	1	2	2	2	1	2	3	2	2	1	2	1	2	1	2	2	2	2	2	2	2	2	2
189	Songlong Village	2	2	2	2	2	2	2	2	2	2	2	2	2	2	2	2	2	3	2	2	2	1	2	2	2	2	2	1	1	3
190	Wangjia Village	3	2	3	2	2	2	2	2	2	2	2	2	2	2	2	2	2	2	2	2	2	2	2	2	2	2	2	2	3	2
191	Paimo Village	2	2	2	2	2	2	2	2	3	2	2	2	2	2	2	2	2	2	2	2	2	2	3	3	2	2	2	2	2	2
192	Gaoyao Village	2	3	3	3	3	3	4	2	2	3	3	4	3	3	3	2	2	2	3	3	3	3	3	4	3	3	3	3	3	4
193	Liujia Village	2	2	1	2	2	1	2	2	2	2	2	2	2	2	2	2	1	2	1	2	2	3	2	2	2	1	2	2	3	3
194	Fenghe Village, La	2	1	2	1	2	1	1	1	1	1	2	2	1	1	1	1	1	2	2	2	2	1	2	2	2	1	2	1	1	2
195	Dahuashui Village,	2	2	3	2	2	2	2	1	2	2	2	1	2	2	2	1	1	2	2	2	2	2	2	3	3	3	2	1	2	2
196	Shuifu Village, Sh	1	2	2	2	2	2	2	2	2	2	2	2	2	2	2	2	2	2	2	1	2	3	2	2	3	2	2	2	2	2
197	Dongmeng Zhai	3	3	3	2	2	3	3	3	2	3	2	3	2	3	2	3	2	2	3	3	3	3	3	4	3	3	3	3	2	3
198	Zenlei Village	2	2	2	2	2	2	2	2	2	2	2	2	2	2	2	2	2	2	2	2	2	2	2	2	2	2	2	2	2	2
199	Shuige Village	1	1	2	2	1	1	1	2	2	1	2	1	2	1	1	2	1	2	2	1	2	2	2	2	1	1	2	1	2	2
200	Laishu Village	2	1	2	2	2	2	2	2	2	2	2	2	2	2	2	2	3	3	2	2	2	2	2	2	2	2	1	2	1	1
201	Paishao Village	2	1	2	2	2	1	2	2	2	3	2	2	2	3	2	2	2	2	2	2	2	2	2	1	3	2	2	2	2	3
202	Yeji Gu Zhai	2	2	3	2	2	3	1	2	2	2	2	2	2	2	2	2	2	2	2	1	2	2	2	2	2	2	2	2	2	2
203	Shibanzhai	2	2	2	2	2	2	2	2	1	2	2	2	2	2	2	2	2	2	2	1	2	2	2	1	2	2	2	2	2	2
204	Geduo Miao Zhai	1	1	2	2	2	1	2	2	2	1	1	1	1	1	2	1	1	1	2	1	2	1	2	2	1	1	2	1	1	1
205	Nanlong Gu Zhai	2	2	2	2	2	2	2	2	1	2	2	3	2	1	2	2	3	2	2	2	2	2	2	2	2	2	3	2	1	2
206	Luyinhe Community	2	2	2	2	2	2	2	3	2	2	2	3	2	2	2	2	3	3	3	3	2	2	2	2	2	2	2	2	2	2
207	Sanglang Village	3	3	3	2	2	2	2	2	3	3	3	3	3	2	2	3	4	3	2	3	2	3	3	2	2	3	2	3	2	3
208	Ganlai Village	2	1	2	2	2	1	1	1	2	1	1	1	1	1	2	1	1	2	1	1	2	1	2	2	1	1	2	2	1	1
209	Zhexiang Village	3	2	2	3	2	4	2	3	3	3	3	2	3	4	2	2	3	2	3	3	2	3	2	2	3	3	3	2	3	4
210	Dazhai Village	3	2	3	3	3	2	3	1	2	3	2	2	2	2	2	2	2	1	2	1	2	1	3	3	2	2	2	2	3	3
211	Weipang Village	1	1	1	2	1	1	1	2	2	1	2	1	2	1	2	2	2	2	2	1	1	1	1	1	1	2	1	1	1	1
212	Bapan Village	3	2	3	3	2	3	3	2	3	3	3	3	3	3	2	3	3	2	3	3	3	3	3	2	3	3	2	4	3	2
213	Lianxin Village	3	3	2	3	2	3	3	2	2	3	3	2	3	4	2	2	2	2	2	3	2	3	3	3	2	3	2	3	3	3
214	Xiaopo Village	2	3	2	2	2	2	3	2	2	1	2	2	2	2	3	2	2	2	2	2	2	2	2	1	2	2	2	1	2	3
215	Damu Village	1	2	2	2	2	2	2	2	2	2	2	2	2	2	2	2	2	2	2	2	2	1	2	2	2	2	3	2	2	2
216	Haiga Village	2	2	2	2	2	2	2	2	2	2	3	3	2	2	3	2	2	2	3	3	1	2	2	3	2	1	2	2	2	1
217	Tianmen Village	2	2	2	2	3	2	2	2	2	3	2	2	2	2	2	2	2	2	2	2	2	2	3	2	2	2	2	2	3	3
218	Yule Village	1	1	2	2	2	1	2	2	2	2	2	1	1	1	1	1	1	2	1	1	1	2	1	1	2	1	2	1	2	1
219	Yanbo Village	2	2	2	2	2	2	1	2	2	2	2	2	2	2	2	2	2	2	2	1	2	1	2	2	2	2	2	3	2	2
220	Xiling Village	2	1	2	2	2	1	2	2	2	2	2	2	2	1	2	2	2	2	2	2	2	1	2	2	2	3	2	2	2	1
221	Shimei Village, Wu	3	3	2	3	2	3	2	2	2	2	3	3	4	4	3	2	3	3	3	2	3	3	2	3	2	3	3	3	3	3
222	Xinjian Community,	3	2	3	1	2	1	2	2	2	2	3	3	2	2	3	2	3	2	2	1	2	2	2	3	2	2	2	2	2	1
223	Meijiang Village	2	2	2	2	1	2	2	2	2	2	2	2	2	2	2	2	2	2	2	2	2	2	2	2	2	2	2	2	2	2
224	Simian Community,	3	2	2	2	2	2	1	2	2	3	2	2	2	2	2	2	3	1	2	2	2	2	2	2	2	2	2	2	2	1
225	Longhai Village	2	2	3	2	2	3	3	2	2	2	2	2	3	2	2	2	2	3	2	3	2	2	2	2	2	2	2	2	2	2
226	Xingfu Village	2	2	2	2	2	2	2	2	2	2	2	2	2	3	2	2	1	2	2	1	3	2	2	2	1	2	2	2	3	2
227	Mama Ya Village	1	2	2	2	2	2	2	2	2	2	2	2	2	2	2	1	1	1	2	1	2	1	2	2	2	3	2	2	2	1
228	Shakan Village	2	2	2	2	2	2	1	2	2	3	2	2	2	2	2	2	2	2	2	2	3	3	2	2	2	3	2	2	3	2
229	Hongyuan Village	3	3	3	2	2	2	4	3	3	2	3	3	3	3	2	2	3	3	3	3	2	3	3	3	3	3	2	3	2	2
230	Jiuling Village	1	1	2	2	2	2	1	2	2	1	2	1	1	1	1	1	2	1	2	1	1	1	1	1	2	1	2	1	2	2
231	Yangchang Village	2	3	2	2	2	1	3	2	2	2	2	2	1	2	2	2	3	3	2	2	2	2	2	2	2	2	3	3	2	2
232	Qianshun Village	3	3	2	3	3	4	3	3	3	4	3	3	2	3	3	2	3	2	2	2	2	3	3	3	3	3	3	4	3	3
233	Tunshang Village	1	1	1	2	1	1	2	2	1	1	2	1	1	1	1	2	1	1	1	1	1	1	1	2	2	2	2	1	1	1
234	Qiangganyan Villag	2	2	3	2	2	3	3	2	2	2	3	1	2	2	1	2	1	2	2	2	2	2	2	1	2	1	2	2	2	2
235	Nongchang Communit	1	2	2	3	2	2	2	2	2	2	2	3	1	3	2	2	2	3	2	2	2	1	2	2	2	3	3	2	2	3
236	Zhongzhai Village	1	1	1	2	2	1	1	1	2	1	1	1	1	1	2	2	1	1	2	1	2	1	2	1	1	2	2	1	2	2
237	Fada Village	2	2	2	2	3	1	3	2	3	3	2	3	2	2	2	2	1	2	2	3	2	1	2	2	2	3	2	3	2	2
238	Caomen Village	2	2	2	2	2	2	2	2	2	1	2	2	1	3	2	2	2	2	2	2	2	3	3	2	1	1	2	2	3	2
239	Dayan Village	3	2	2	2	2	3	3	3	3	3	3	4	3	3	2	3	3	3	3	3	3	3	3	4	3	3	2	3	2	3
240	Lula Village	2	3	2	2	2	2	3	2	1	1	2	2	2	2	2	2	3	3	2	2	2	2	2	2	2	2	2	2	3	3
241	Yelang Village	2	2	2	2	2	2	2	2	2	2	2	3	2	1	2	2	3	3	3	2	2	1	2	2	2	2	2	3	3	3
242	Tuping Community	3	2	2	2	2	2	2	3	2	2	2	2	2	2	2	2	2	2	2	2	2	2	2	2	2	2	2	1	1	1

243	Bamei Village	2	2	3	2	3	2	2	2	2	2	2	2	1	2	1	2	2	1	2	1	2	2	2	1	2	2	2	2	2	3
244	Sidu Village	2	2	2	2	2	2	2	2	2	2	2	2	2	2	2	2	2	2	2	2	2	2	2	2	2	2	2	2	2	2
245	Liaojia Tun Village	2	2	2	2	3	2	3	2	3	2	2	2	2	2	2	2	3	2	2	2	2	2	2	2	2	2	2	2	2	1
246	Longmen Village	3	2	2	2	2	2	2	2	3	2	2	2	2	3	2	2	2	2	2	2	2	3	2	2	2	2	2	2	2	2
247	Laozhai Village	4	4	3	2	3	2	3	3	3	3	3	3	3	3	2	3	3	2	3	3	3	3	2	3	3	3	3	3	3	3
248	Xieqiao Village	2	2	2	2	2	2	3	3	2	2	2	2	2	2	2	3	2	2	2	2	2	2	2	1	1	2	2	2	2	2
249	Fangjialing Village	3	3	2	3	2	4	2	2	2	3	2	3	3	3	2	3	4	3	3	3	3	3	2	3	3	3	3	3	3	3
250	Xiaba Village, Hulu	2	3	3	2	2	2	3	2	2	2	2	2	3	2	2	2	2	2	2	2	2	2	2	3	2	3	2	2	2	1
251	Cenduo Village	2	1	3	2	2	3	2	2	2	2	2	2	2	1	2	2	2	2	2	2	2	2	2	2	2	2	2	2	2	2
252	Fengyan Village	2	2	2	2	2	2	2	2	2	3	2	1	2	1	3	2	2	2	2	3	3	2	2	2	2	2	2	2	2	2
253	Yechang Village	3	3	3	3	3	3	3	2	2	3	3	3	3	3	2	2	3	2	3	3	3	2	3	4	2	4	3	3	3	3
254	Baqiao Village	1	2	2	2	2	2	2	1	2	2	2	2	2	3	2	2	3	3	2	2	3	2	3	2	2	2	2	2	1	1
255	Poqi Village	2	3	2	2	2	1	2	2	2	2	2	2	1	2	2	2	2	2	2	2	2	2	2	2	2	2	2	2	2	1
256	Huajiang Village	2	2	3	2	2	2	3	2	2	3	2	2	2	2	1	2	2	2	2	2	2	2	2	2	2	2	1	2	2	2
257	Nama Village	2	2	2	3	2	2	2	2	2	3	2	2	2	2	2	3	2	1	2	2	1	2	2	1	2	1	2	2	2	3
258	Dongqing Village	2	2	2	2	2	1	1	2	2	1	2	1	1	1	2	2	2	1	2	1	2	1	2	2	1	1	1	1	1	1
259	Xintun Village	2	2	2	2	2	2	2	2	3	2	2	2	2	3	3	2	2	2	3	2	2	2	2	3	2	2	2	2	2	2
260	Youmai Village	1	2	1	2	2	1	1	2	2	1	1	1	1	1	1	2	1	2	2	2	1	2	2	1	2	2	2	1	2	1
261	Fuyao Village	1	1	2	1	2	1	1	2	1	2	2	1	1	1	2	2	1	1	1	2	2	1	2	1	2	2	1	2	2	1
262	Lishu Village	2	2	2	2	2	3	2	2	2	2	2	2	1	2	3	2	3	2	2	2	2	1	2	2	2	2	2	2	2	2
263	Mandong Village, Q	1	2	2	1	2	2	2	2	1	1	3	2	1	2	2	2	2	2	2	2	2	3	2	2	3	2	2	3	2	1
264	Tunzai Village	3	3	2	2	2	2	2	2	3	2	2	2	2	2	2	2	2	2	2	2	2	1	2	2	2	3	2	2	2	1
265	Gunshui Village	2	2	2	2	2	2	2	2	2	2	2	1	1	2	2	2	2	2	2	3	2	1	2	2	2	2	2	2	2	1
266	Xiaowengxi Village	2	2	2	2	2	1	3	2	2	2	2	2	2	2	1	2	2	2	2	2	2	2	2	2	2	1	1	1	1	2
267	Longjiao Village	2	2	2	2	2	2	2	2	2	1	1	1	2	1	2	2	2	2	2	2	2	2	2	2	2	3	2	2	2	2
268	Yazhong Village	2	1	2	1	2	3	2	2	2	2	1	1	2	2	2	2	2	2	2	2	2	2	2	2	2	2	2	2	1	2
269	Fengshou Village, M	1	3	2	2	2	2	2	2	2	2	2	2	2	2	2	2	2	2	2	2	2	1	2	2	2	2	2	2	2	3
270	Zhouping Village	2	2	2	2	2	2	2	2	2	2	2	2	2	2	1	2	2	3	3	1	2	3	3	2	3	2	2	2	3	2
271	Longtang Village	2	2	2	2	2	2	3	2	2	2	2	2	3	2	2	1	1	2	2	1	2	2	3	2	2	3	2	2	2	2
272	Gonghe Village, Gar	1	1	2	1	3	2	1	2	2	3	2	1	1	1	2	2	2	1	1	1	2	1	2	1	2	1	1	1	1	1
273	Xiateng Village	3	3	2	3	3	3	3	2	2	3	2	3	2	4	2	3	3	3	2	2	2	3	2	3	3	3	2	3	3	3
274	Huazhai Village	2	2	2	2	2	3	2	2	2	1	2	2	2	2	3	2	2	2	2	2	2	2	3	3	2	2	3	2	2	3
275	Zhaio Village	2	2	2	2	2	2	2	2	2	2	3	2	3	2	2	3	3	2	2	1	2	2	2	1	2	1	2	2	2	2
276	Pingqiu Village	2	2	2	2	2	2	2	3	2	1	3	2	3	2	1	2	1	2	2	1	2	3	3	2	2	2	3	2	2	2
277	Huangmen Village	3	2	2	2	2	2	2	2	2	1	3	2	2	2	2	2	2	3	2	2	2	1	2	2	2	3	2	3	2	2
278	Xiaoguang Village	2	2	2	2	2	2	2	2	2	2	3	2	2	3	2	3	3	2	2	2	2	2	2	2	2	2	2	2	2	2
279	Fangzhao Village	2	4	3	2	2	3	3	2	3	2	3	3	2	3	2	3	2	2	2	3	2	3	3	3	2	4	2	2	3	2
280	Jiaomi Village	3	2	2	2	2	2	2	2	2	3	2	3	2	1	2	2	3	2	2	2	2	2	2	2	2	2	2	1	2	2
281	Jiubai Village	2	2	2	2	2	2	2	2	2	2	2	1	2	2	1	2	3	2	2	1	3	3	3	3	3	2	2	2	3	2
282	Jiuzhai Village	2	3	2	2	2	2	2	2	2	2	2	2	2	2	2	2	2	2	2	2	2	2	2	2	2	3	2	2	2	2
283	Wanzhai Village	1	2	2	2	2	2	2	2	2	2	2	2	1	2	3	2	1	2	2	3	2	2	3	2	2	2	2	3	2	2
284	Gunzong Village	2	2	2	2	2	1	1	2	2	3	2	2	2	2	2	2	2	2	2	2	2	2	3	2	3	2	2	3	2	3
285	Shang’ao Village	2	1	2	3	2	2	2	2	2	1	2	3	3	1	2	2	2	2	3	2	2	2	3	2	3	2	2	3	2	2
286	Qingshan Village	2	2	2	1	2	2	2	2	2	1	2	2	2	2	2	2	2	2	2	2	2	3	2	2	2	1	2	2	2	2
287	Heba Village	2	3	2	2	2	2	1	2	2	3	2	3	2	2	2	2	2	3	2	2	2	1	2	2	2	3	2	2	2	2
288	Wengpao Village	2	2	2	2	2	2	1	2	2	2	2	2	1	2	2	2	2	2	2	2	2	2	2	2	3	2	2	2	2	2
289	Qingjiang Village	1	2	2	2	2	2	2	1	2	1	2	1	2	2	2	2	3	2	2	1	2	2	2	2	2	2	2	2	2	2
290	Gaozhai Village	2	3	3	2	2	3	2	2	2	4	3	4	3	3	3	3	2	2	3	3	3	3	2	3	2	3	2	3	2	3
291	Yangxian Village	1	2	2	2	2	2	1	3	2	2	2	3	2	2	3	2	1	2	2	2	2	2	2	2	3	3	2	2	2	2
292	Wuzuo Village	2	2	2	2	2	2	2	2	2	2	2	1	2	2	2	2	2	2	2	3	2	2	2	2	2	1	1	1	2	2
293	Laodong Village	1	1	1	1	2	1	1	2	2	1	1	1	1	1	2	1	1	1	1	1	1	2	2	1	2	1	1	1	2	1
294	Jialao Village	3	3	2	3	3	3	3	2	2	4	2	4	3	2	3	2	4	3	3	3	3	2	3	2	2	3	3	3	3	4
295	Pingyang Village, Z	3	4	3	3	2	3	3	3	2	3	3	3	3	3	2	3	4	3	2	3	3	4	3	3	3	4	2	3	3	3
296	Jinfeng Village	2	3	2	2	2	3	3	3	3	3	3	4	3	4	3	3	2	3	3	3	2	3	3	3	2	3	3	2	3	4
297	Xinqiaoying Village	1	1	2	2	2	2	1	1	2	1	2	2	1	1	2	2	1	2	1	2	2	1	1	1	2	2	2	1	2	1
298	Shuipu Village	2	2	2	2	2	2	2	2	2	2	2	2	2	2	2	2	2	2	3	2	2	2	2	1	2	2	2	3	2	3
299	Datumiao Zhai	2	3	2	2	3	2	3	2	2	2	2	2	1	1	2	2	2	2	2	2	2	2	2	2	2	1	1	1	2	2
300	Juegong Village, Ba	2	1	2	2	2	2	3	2	2	1	3	2	2	2	2	2	2	2	2	2	2	3	3	2	2	1	2	1	2	2
301	Jingzhou Village	2	1	2	2	2	2	2	2	1	2	2	3	3	2	2	3	2	2	2	2	2	3	2	2	2	1	2	2	2	2
302	Mahai Village	2	2	2	2	3	3	2	2	3	2	2	2	1	3	2	2	2	2	2	2	2	2	2	2	2	1	2	2	2	2
303	Honghe Village	2	3	2	3	2	3	2	2	2	3	3	4	2	3	2	2	3	3	3	3	3	3	3	2	3	3	3	4	2	3
304	Zhongyuan Village	3	4	2	3	3	2	3	3	3	4	2	3	3	3	2	2	3	2	3	2	3	4	3	4	3	3	3	3	3	3
305	Zouma Village, Kong	2	2	3	2	2	2	2	2	2	3	2	1	2	1	2	3	2	1	2	2	2	2	2	2	2	2	2	2	3	2
